# Multi-Population Classical HLA Type Imputation

**DOI:** 10.1371/journal.pcbi.1002877

**Published:** 2013-02-14

**Authors:** Alexander Dilthey, Stephen Leslie, Loukas Moutsianas, Judong Shen, Charles Cox, Matthew R. Nelson, Gil McVean

**Affiliations:** 1 Department of Statistics, University of Oxford, Oxford, United Kingdom; 2 Wellcome Trust Centre for Human Genetics, Oxford, United Kingdom; 3 Murdoch Childrens Research Institute, Royal Children's Hospital, Parkville, Victoria, Australia; 4 Quantitative Sciences, GlaxoSmithKline, Research Triangle Park, North Carolina, United States of America; 5 Quantitative Sciences, GlaxoSmithKline, Stevenage, United Kingdom; University of Washington, United States of America

## Abstract

Statistical imputation of classical HLA alleles in case-control studies has become established as a valuable tool for identifying and fine-mapping signals of disease association in the MHC. Imputation into diverse populations has, however, remained challenging, mainly because of the additional haplotypic heterogeneity introduced by combining reference panels of different sources. We present an HLA type imputation model, HLA*IMP:02, designed to operate on a multi-population reference panel. HLA*IMP:02 is based on a graphical representation of haplotype structure. We present a probabilistic algorithm to build such models for the HLA region, accommodating genotyping error, haplotypic heterogeneity and the need for maximum accuracy at the HLA loci, generalizing the work of Browning and Browning (2007) and Ron et al. (1998). HLA*IMP:02 achieves an average 4-digit imputation accuracy on diverse European panels of 97% (call rate 97%). On non-European samples, 2-digit performance is over 90% for most loci and ethnicities where data available. HLA*IMP:02 supports imputation of HLA-DPB1 and HLA-DRB3-5, is highly tolerant of missing data in the imputation panel and works on standard genotype data from popular genotyping chips. It is publicly available in source code and as a user-friendly web service framework.

## Author Summary

The human leukocyte antigen (HLA) proteins influence how pathogens and components of body cells are presented to immune cells. It has long been known that they are highly variable and that this variation is associated with differential risk for autoimmune and infectious diseases. Variant frequencies differ substantially between and even within continents. Determining HLA genotypes is thus an important part of many studies to understand the genetic basis of disease risk. However, conventional methods for HLA typing (e.g. targeted sequencing, hybridisation, amplification) are typically laborious and expensive. We have developed a method for inferring an individual's HLA genotype based on evaluating genetic information from nearby variable sites that are more easily assayed, which aims to integrate heterogeneous data. We introduce two key innovations: we allow for single HLA types to appear on heterogeneous backgrounds of genetic information and we take into account the possibility of genotyping error, which is common within the HLA region. We show that the method is well-suited to deal with multi-population datasets: it enables integrated HLA type inference for individuals of differing ancestry and ethnicity. It will therefore prove useful particularly in international collaborations to better understand disease risks, where samples are drawn from multiple countries.

## Introduction

Statistical imputation of classical human leukocyte antigen (HLA) alleles from SNP genotypes in case-control studies has become established as a valuable tool for identifying and fine-mapping signals of disease association in the MHC. Application of the HLA type imputation framework HLA*IMP [1], [2] has, for example, helped to fine-map secondary HLA-based risk effects in multiple sclerosis [3], contributed to characterizing an HLA-related gene-gene interaction in psoriasis [4], and was essential in refuting a suspected strong HLA contribution to childhood B-cell precursor acute lymphoblastic leukaemia [5]. Classical HLA allele imputation has, in other settings, been used to identify particular amino acids within classical peptides contributing to disease risk [6].

Classical HLA allele imputation is complicated by hyperpolymorphism (*HLA-B* , for example, has dozens of common alleles and 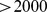 rare alleles) and the complex haplotype structure of the HLA region, justifying the development of specialized imputation machinery. Linkage disequilibrium (LD) between loci usually declines with distance, as LD is broken down by recombination. In the HLA, however, this is not always empirically true. Many comparatively distant SNPs carry information on the allelic state of the classical HLA genes [7]. Fully capturing this information is not trivial. For example, a commonly used model in statistical genetics, the Li and Stephens approximation [8], does not allow for explicit modelling of long-distance LD relationships due to its reliance on a first order Markov chain. HLA*IMP therefore uses a particular formulation of the Li and Stephens approximation that assigns equal weight to all selected SNPs irrespective of distance from the classical locus of interest [2]. We have since demonstrated (e.g., [1]) that this formulation leads to highly accurate HLA type imputations, at least when reference and imputation panel are derived from the same population.

For the increasingly important use case of multi-population studies (where the reference and analysis panels consist of samples taken from multiple, possibly diverse, populations), HLA type imputation has, however, remained challenging: Imputation accuracy is limited by the extent to which the reference panel captures the diversity of the target population and current methods typically rely on single-source reference panels of Northern European origin [1], [9].

The obvious solution, successfully applied in SNP genotype imputation [10], [11], [12], is to make use of diverse multi-population reference panels. However, an additional challenge of multi-population classical HLA type imputation is that single HLA alleles can appear on multiple SNP haplotype backgrounds [7], a phenomenon we refer to as “haplotypic heterogeneity”. Moreover, genetic data obtained from multiple data sets from different populations is likely to contain systematic genotyping artefacts. Here we present HLA*IMP:02, an HLA type imputation method that is particularly aimed at inference in multi-population and multi-ethnicity settings. That is, it is designed to accommodate both haplotypic heterogeneity and genotyping error.

Inference under HLA*IMP:02 is based on a graphical model of the haplotype structure of the MHC region. We motivate this choice by restating an observation made by Browning and Browning [13]: Graphical haplotype models are well-suited to model LD relationships spanning different scales of distance (“variable-length Markov chains”), which fits with the HLA region's empirically observed LD structure. We present an algorithm to build such models from a set of reference genotype data. The main design features of the algorithm are that it takes into account haplotype uncertainty introduced by potential genotyping error, that it allows for haplotypic heterogeneity and that it tailors the graphs to make them maximally informative about the allelic state of the HLA loci. Our algorithm can be viewed as a probabilistic generalization of the works of Browning and Browning [14]. Compared with HLA*IMP, HLA*IMP:02 also offers a couple of practical advantages: it is highly tolerant of missing data in the inference panel and supports imputation of HLA-DPB1 and HLA-DRB3-5.

It is instructive to explicitly consider how the design of HLA*IMP:02 leads to an improved ability to deal with heterogeneous data, as compared to HLA*IMP:

- Data representation: HLA*IMP:02 builds a combined locus-specific haplotype graph model of the whole dataset. In HLA*IMP, in contrast, reference genotype data is phased and separated by HLA alleles. All further steps are based on these allelic groups (one for each HLA allele in the reference panel). This design prevents HLA*IMP from sharing SNP haplotype information across haplotypes carrying different HLA alleles.
- Maximising imputation performance: HLA*IMP:02, uses all available SNPs in the HLA region. However, while building the haplotype graph no two internal haplotype states that exhibit different association patterns to HLA alleles are combined, thus maintaining accuracy specifically for HLA allele prediction. HLA*IMP, in contrast, carries out a process of SNP selection, identifying SNPs in the region that are informative for accurate prediction of HLA types. Finding a set of consistently informative SNPs becomes increasingly difficult as the degree of stratification in the reference panel increases.
- Inference model: In the haplotype-graph approach of HLA*IMP:02, haplotypes are not grouped in advance. If an allele appears on multiple SNP haplotypes, there will be multiple paths through the graph leading to the allele. Inference is based on comparing the likelihoods of all possible paths. Ambiguity therefore typically only arises if two or more alleles share the same SNP haplotypes, but not if one allele appears on more than one background. Additional heterogeneity in the reference panel (characterized by alleles appearing on more than one unique background) does not decrease the model's ability to correctly infer HLA genotypes. HLA*IMP, in contrast, appears to suffer decreased performance in both scenarios (one allele/multiple backgrounds, multiple alleles/one background). This is perhaps because inference under HLA*IMP is based on finding the most similar *group* of haplotypes (implemented through a particular formulation of the Li and Stephens [8] Hidden Markov Model, HMM). Additional heterogeneity in an allele's SNP background necessarily reduces group-wise average similarity and dilutes the model's ability to correctly infer HLA genotypes.

We carry out three experiments to investigate the performance of HLA*IMP:02 on reference panels of varying heterogeneity. In the first experiment, we apply HLA*IMP:02 to a homogeneous (predominately British) reference panel and show that it performs as well as HLA*IMP in this baseline scenario. In the second experiment, we demonstrate that HLA*IMP:02 achieves high imputation accuracy at 4-digit HLA type resolution (reflecting primary sequence of the HLA proteins) when applied to an integrated cross-European reference panel, clearly outperforming HLA*IMP. In the third experiment, we use a highly heterogeneous multi-ethnic reference panel to impute HLA genotypes of Asian, African-American, African, European and Hispanic individuals. We show that accuracy for the European individuals remains essentially unchanged by making the reference panel more heterogeneous and that the model achieves high imputation accuracy for the other ethnicities at 2-digit resolution, which reflects the serological properties of the HLA alleles (see Subsection “Validation” for a precise definition in our context).

## Materials and Methods

### HLA*IMP:02

**Figure 1:**
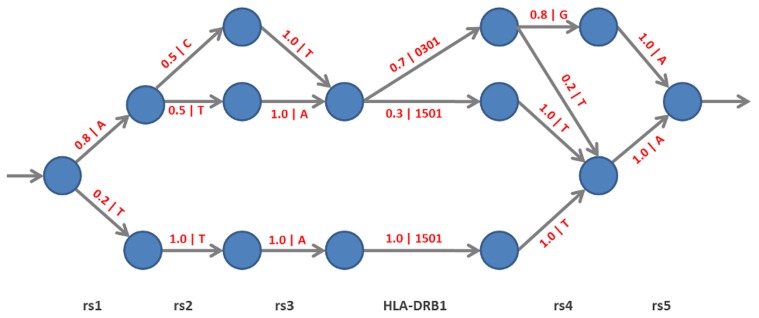
Features of haplotype graph models. Illustration of the features of haplotype graph models. Haplotype graphs are a subclass of connected directed graphs and belong to the class of acyclic probabilistic finite automata. Their most important properties are illustrated here: 1) They are leveled, i.e. each vertex 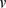 has an associated positive number 1, and all edges emanating from 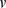 at level 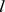 lead to a vertex at level 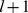 and represent the same genetic locus. Vertices at level 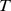 are final vertices with no outgoing edges, and there is a path from every vertex in the graph to one of the final vertices. 2) Edges carry “emission symbols” which are emitted when an edge is traversed (in the figure: the symbols after the “|” character adjacent to the edges), and there are no two edges emanating from the same vertex which carry the same symbol. 3) Each vertex has an edge probability distribution over its attached edges (in the figure: the numbers in front of the “|” character adjacent to the edges), according to which an edge is selected conditional in being at that vertex.

We use an acyclic probabilistic finite automaton (“haplotype graph”, see Figure 1) to represent haplotype structure in the HLA region [14], [15]. The haplotype graph describes the haplotype structure of SNPs around the classical HLA loci. In Figure 1, each possible path through the graph also passes through an edge carrying an HLA allele, and therefore specifies a corresponding HLA genotype. The likelihood of any particular path depends on the branching structure of the graph (as specified by the probabilities on the edges in Figure 1) as well as on the observed SNP genotypes from an individual that we want to make inference for. For example, if we observe the SNP genotypes TTA?TA (the question mark stands for the unknown HLA allele, and we only consider the haploid case here for simplicity), the likelihood of the path passing through the bottom nodes (and the 1501 allele) is 0.2, and the likelihood of all others paths is 0 (not allowing for any deviations from the edge labels for the sake of this argument). For ATA?GA, the case is also clear: 0301 is the only possible allele. If we now change the second-last genotype to T (yielding ATA?TA), there are two possible paths. The one passing through 1501 has a likelihood of 0.012, and the other one passing through 0301 has a likelihood of 0.056. Conditional on the observed SNP genotypes, 1501 is therefore approximately twice as probable as 0301. Changing the second and third genotypes would not influence this result (which relates back to our introductory comments on the variable length of captured LD relationships: the first position influences inference, the second and third do not).

In order to use haplotype graphs for imputation, there are two general problems to address: how to construct a haplotype graph from a set of reference data, and how to use an existing graph for imputing the genotype of an additional individual. Methods to construct and use haplotype graph-like objects from a set of reference data were discussed by Ron et al. [15] and introduced into the field of statistical genetics by Browning [13] and Browning and Browning [14]. The work we present here can be viewed as a probabilistic generalization of the works of Ron et al. [15] and Browning and Browning [14]. To use haplotype graph models specifically for HLA type inference, we have developed solutions to two related tasks: how to build a haplotype graph model from the reference panel allowing for errors in SNP genotype data and haplotypic heterogeneity and how to boost accuracy for HLA allele imputations. A full and formal description of the HLA*IMP:02 algorithm can be found in the Supporting Text S1. Here we provide outline of our algorithm and the inference process, highlighting where we generalized and extended previous approaches.

Constructing a haplotype graph from a set of reference data (including both SNP and HLA genotypes) is an iterative process, consisting, as in BEAGLE, of three main steps:

- Initialization: for each individual, populate the set 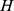 of current haplotype estimates by sampling from the uniform distribution over all genotype-consistent haplotype pairs. In contrast to BEAGLE, we preserve missing data in the generated haplotype pairs.
- Probabilistic graph construction: build a haplotype graph object from the set 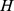 of current haplotype estimates. Each element in 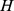 corresponds to one path through the graph which is going to be constructed. We define a probability distribution over possible paths for each element in 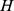 and probabilistically attach the elements in 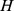 to nodes in the graph. This enables us to allow for genotyping errors and missing data in 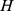 and puts some part of the probability mass of similar haplotypes on the same nodes, even if they differ in single positions (by setting the probability of genotyping error to 0, one obtains the deterministic BEAGLE/Ron et al. [15] mode of haplotype propagation through the graph). In the process of building the graph, we collapse similar nodes for reasons of parsimony and computational efficiency. In defining node similarity, we introduce criteria that relate to each node's pattern of association with the HLA loci along the graph, and prevent collapsing two nodes that exhibit differing patterns of LD with HLA alleles (by setting the set of the loci that these additional criteria apply to the empty set, one obtains the conventional similarity criterion from BEAGLE/Ron et al. [15]).
- Resampling: Construct the diploid HMM induced by the constructed haplotype graph and re-populate 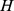. If a predefined number of iterations has not been exceeded, fit this HMM to the reference genotype data, re-populate 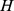 with haplotype samples from the HMM (imputing missing data) and go to step 2. Like Browning and Browning [16], we use an HMM that allows for genotyping error.

The HMM resulting from the final iteration is used to generate HLA type estimates for all following imputation operations (BEAGLE, in contrast, builds joint haplotype graphs of imputation and reference panels, and carries out imputation as part of this procedure, which requires special measures for assuring convergence if the joint set is dominated by samples from the imputation dataset).

### Availability, Performance, Usability

Source code for HLA*IMP:02 is available from http://oxfordhla.well.ox.ac.uk (free for academic use). Compiling and running the program requires a standard UNIX server environment (ideally with multiple CPU cores and 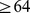 GB RAM).

To give an idea of the expected runtime, producing the graph for *HLA-A* for the first experiment presented in the “Results” section took approximately 137 CPU hours (user plus system time for a single CPU; the program supports parallelization via openMP, so that the actual runtime on modern multi-CPU systems is much lower); carrying out inference for a single individual required approximately 4 CPU seconds (user plus system time).

**Figure 2:**
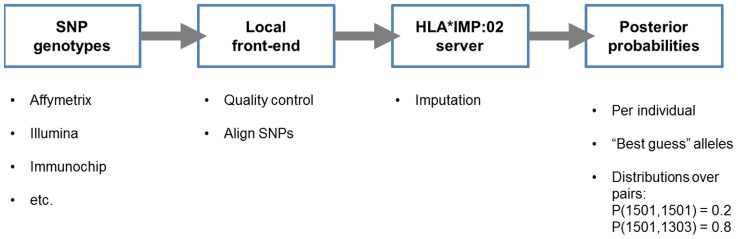
Standard workflow for HLA*IMP:02. Standard workflow for HLA*IMP:02: standard output data from popular genotyping platforms, for example current Illumina or Affymetrix chips, are converted into the HLA*IMP format using the locally installed front-end program. The front-end also carries out necessary steps of quality control, such as aligning SNP strandedness. The output files from the front-end are submitted to the HLA*IMP:02 server, which processes the data and produces imputations (posterior probabilities over pairs of alleles as well as a “best guess” pair of two alleles with associated quality scores).

Like HLA*IMP, HLA*IMP:02 is also available as a front-end/back-end web service that integrates data preparation, QC and imputation. Figure 2 shows the steps typically required to produce HLA type imputations, starting from SNP genotypes (for example in PLINK [17], CHIAMO [18] or VCF formats). The system supports virtually all currently employed genotyping platforms, including genotyping arrays from Affymetrix, Illumina, and the Immunochip. The front-end converts genotype data into the format used by HLA*IMP:02, carries out quality control based on data completeness and aligns SNP genotypes to the positive strand (as defined in HapMap). All output data from the front-end can be directly uploaded to the HLA*IMP:02 server. Run in standard mode, the HLA*IMP:02 back-end will also produce allele- and locus-specific cross-validation estimates of accuracy, specific to the SNPs available in the user dataset. To ensure data protection and security, sample identifiers have to be anonymized prior to submission. The server stores all user data in a specially protected area, with no read access for the normal web server processes. Upon completion of an imputation job, the server generates a secondary access key, which is directly sent to the user; only the combination of access key and user account password will enable access to the imputation results.

### HLA*IMP:01

We compare the performance of HLA*IMP:02 to HLA*IMP, which we refer to as “HLA*IMP:01” for clarity. HLA*IMP:01 has been described elsewhere [1], [2]. Windows of 400 SNPs around the classical HLA loci and population prior frequencies, estimated from the reference panel, for classical HLA alleles were found to give good results, and these settings are identical to those used by the Internet implementation of HLA*IMP (http://oxfordhla.well.ox.ac.uk) and those used for recent genome-wide association studies [3], [4], [19].

### Validation

We validate HLA type imputations at the genotype level in a locus-specific manner, i.e. compare two unordered sets with two elements each for each individual and locus, one set (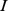) representing the imputation results and the other (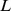) containing the lab-derived types. We only consider individuals who carry two HLA alleles typed at 4-digit resolution at the locus under validation or one allele at 4-digit resolution and one missing allele. For 2-digit (serological properties of the HLA alleles) validation, we consider the same individuals, but we set the last 2 digits of each HLA allele to ‘00’ (this will lead to an underestimate of accuracy in some cases, as there are some serologically defined 2-digit allele groups that map to more than one pair of leading two digits). We may or may not apply a posterior probability call threshold 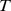 on the per-allele level (see Section “HLA type inference” of the Supporting Text S1 for a description of how we calculate allele-specific posterior probabilities) to our imputations before validating.

If there is no missing data in 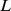, there are three possible cases:

- 0 imputations left after thresholding: we count 0 correctly imputed alleles out of 0.
- 1 imputation (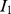) left after thresholding: we count 1 correctly imputed alleles out of 1 if 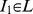, otherwise 0 out of 1.
- 2 imputations left after thresholding: we count 0 correct imputations out of 2 if 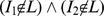, 1 out of 2 if 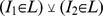, 2 out of 2 otherwise. (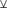 is the “exclusive OR” operator, which is true if and only if exactly one of the arguments is true).

If 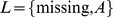 (i.e. only one allele has been typed), there are also three possible cases:

- 0 imputations left after thresholding: we count 0 correctly imputed alleles out of 0.
- 1 imputation (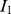) left after thresholding: we count 1 correctly imputed alleles out of 1 if 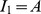, otherwise 0 out of 1.
- 2 imputations left after thresholding: we count 1 correct imputations out of 1 if 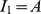 or 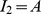 or both.

In terms of thresholding strategies, we use either no threshold; or a threshold of T = 0.7 for both models; or a threshold of T = 0.7 for HLA*IMP:01 (as recommended in Dilthey et al. [1]) and a threshold matched to obtain equal call rates for HLA*IMP:02. The last strategy is only employed to ensure comparability of results for the first baseline experiment (see next section), in which we compare the performance of HLA*IMP:01 and HLA*IMP:02 on a homogeneous dataset.

At the per-locus level, we use concordance (which is, at the per-locus level, identical to PPV) as a measure of accuracy. We also provide more detailed statistics at the allele level (see below).

### Data

The experiments presented in this paper are based on different combinations of two datasets.

The first set, denoted “Golden Set” (GS), has been described elsewhere [1] and comprises 2512 individuals from the 1958 Birth Cohort (http://www.b58cgene.sgul.ac.uk/), the HapMap CEU [20] and the CEPH CEU+ [7] cohorts. Genotyping of the GS was carried out on the Illumina 1.2M and Affymetrix Genome-Wide Human SNP Array 6.0 chips. HLA typing methods vary according to the original cohort. Protocols for 1958 BC HLA genotyping are described online (https://www-gene.cimr.cam.ac.uk/public_data/HLA/HLA.shtml). CEU and CEU+ were typed using exon-sequencing methods.

The second set, denoted “HLARES_ALL”, has been provided by GlaxoSmithKline and comprises (post quality control, as described in Dilthey et al. [1]) 1460 individuals from diverse, though mainly European or European-ancestry, populations (see Supporting Table S2). The individuals in HLARES_ALL were drawn from several clinical trials and typed on the Illumina 1 M SNP genotyping platform, and classical HLA type information (derived by exon sequencing) is available for many of them (see Table 1 for details). Genome-wide principal components analysis (PCA) of the samples in HLARES_ALL was carried out using the program EIGENSTRAT [21].

**Table 1:** Dataset characteristics summary.

Number of individuals with at least one allele typed at 4-digit resolution (2-digit for DRB3, DRB4, DRB5)											
	SNPs	HLA-A	HLA-B	HLA-C	HLA-DQA1	HLA-DQB1	HLA-DRB1	HLA-DPB1	HLA-DRB3	HLA-DRB4	HLA-DRB5
**GS**	7733	1556	1570	1153	87	1585	1517	0	0	0	0
**HLARES_EU**	7568	308	1060	349	279	446	897	74	282	282	282
**GS&HLARES_EU**	6056	1864	2630	1502	366	2031	2414	74	282	282	282
**GS&HLARES_EU 2/3**	6056	1253	1758	1017	250	1359	1592	50	187	187	187
**GS&HLARES_EU 1/3**	6056	611	872	485	116	672	822	24	95	95	95
**GS&HLARES_ALL**	7632	2028	3063	1675	521	2223	2809	112	353	353	353
**GS&HLARES_ALL 2/3**	7632	1356	2055	1129	354	1495	1853	77	242	242	242
**GS&HLARES_ALL 1/3**	7632	672	1008	546	167	728	956	35	111	111	111

Number of 4-digit HLA alleles (2-digit for DRB3, DRB4, DRB5)											
		HLA-A	HLA-B	HLA-C	HLA-DQA1	HLA-DQB1	HLA-DRB1	HLA-DPB1	HLA-DRB3	HLA-DRB4	HLA-DRB5
**GS**		27	46	21	7	18	34	0	0	0	0
**HLARES_EU**		34	60	28	14	17	43	18	5	3	4
**GS&HLARES_EU**		38	65	30	14	19	47	18	5	3	4
**GS&HLARES_EU 2/3**		33	60	27	13	19	42	15	5	3	3
**GS&HLARES_EU 1/3**		28	52	24	11	17	37	13	5	3	4
**GS&HLARES_ALL**		58	110	39	15	21	62	21	5	3	4
**GS&HLARES_ALL 2/3**		48	99	34	14	20	56	17	5	3	4
**GS&HLARES_ALL 1/3**		45	85	30	13	20	49	17	5	3	3

The upper part of this table shows the number of individuals that are available for building the HLA*IMP:02 graphs in a locus-specific manner. For HLA*IMP, the number of available haplotypes is approximately double the individual number. The bottom part of the table shows allelic diversity for all reference and validation datasets used in our study. Note that the allelic diversity in the HLARES and in the GS&HLARES 2/3 datasets is bigger than in the GS.

We resolve ambiguous HLA type information by using the maximum population frequency call. Besides that, we treat all HLA genotypes “as is”; that is, we make no attempt to control, for example, for changes of HLA nomenclature or allele databases. This might lead to slight underestimates of accuracy (in the worst case, we do not recognize identical alleles as identical).

In the first experiment (homogeneous reference), we evaluate the performance of statistical HLA type imputation (*HLA-A* , *-B* , *-C* , *-DQA1* , *-DQB1* and *-DRB1*) on cross-European samples, based on a mainly British reference panel. We use the GS as reference panel to impute classical HLA types of those samples in HLARES_ALL with self-declared European ancestry (HLARES_EU) and measure concordance with lab-derived HLA type information where available. Supporting Table S2 describes the distribution of countries the individuals in HLARES_EU were sampled from. There are 6056 SNPs in the extended MHC region (xMHC, here defined as the chromosomal region on chromosome 6 from position 25,921,129 to position 33,535,328, build 36; see Horton et al. [22]) in the intersection of the GS and HLARES_EU datasets. To mirror the context in which HLA*IMP:01 was applied in recent genome-wide association studies [3], [4], [19], we further restrict the available SNP set to those also present in one of them [3], resulting in 2020 SNPs.

In the second experiment (medium heterogeneity), we evaluate the performance of statistical HLA type imputation on European samples, based on a cross-European reference panel. To obtain a cross-European reference panel (GS&HLARES_EU), we merge the GS and HLARES_EU datasets, keeping only SNPs in the intersection of the two panels (6056 xMHC SNPs). We randomly split GS&HLARES_EU into two panels, and use the first one (GS&HLARES_EU 2/3, containing approximately 2/3 of the original data) as reference, and the second one (GS&HLARES_EU 1/3, approximately 1/3 of the original data) as validation panel. Referring to the increased population structure in GS&HLARES_EU 2/3 as compared to GS, we call GS&HLARES_EU 2/3 a heterogeneous reference panel. We measure concordance with experimentally-derived HLA type information where available. We use the data on additional loci present in GS&HLARES_EU (*HLA-DPB1* , *-DRB3* , *-DRB4* , *-DRB5*) to evaluate how well their allelic states can be imputed. Also, in a variation of the second experiment, we modify the SNP density in the xMHC region to investigate to what extent performance will depend on the selected SNP genotyping platform and data missingness profiles.

In the third experiment (high heterogeneity), we evaluate the performance of statistical HLA type imputation on multi-ethnic samples, based on a multi-ethnic reference panel. To obtain a multi-ethnic reference panel (GS&HLARES_ALL), we merge the GS and HLARES_ALL datasets. We also include the HapMap YRI cohort [20], as individuals self-reporting as of African ancestry constitute a subset of HLARES_ALL. We keep all available SNP genotypes from the intersection of GS and YRI (7733 SNPs from GS of which 7632 xMHC SNPs are also present in YRI), and combine them with the SNP genotypes from HLARES_ALL (6050 SNPs, setting the remaining 1582 SNP genotypes to “missing”). The resulting set GS&HLARES_ALL has 7632 xMHC SNPs. We randomly split GS&HLARES_ALL in two panels, and use the first one (GS&HLARES_ALL 2/3, containing approximately 2/3 of the original data) as reference, and the second one as (GS&HLARES_ALL 1/3, approximately 1/3 of the original data) as validation panel. We call GS&HLARES_ALL 1/3 a “highly heterogeneous” reference panel. Note that GS&HLARES_ALL is still dominated by samples of European origin. We measure concordance with experimentally-derived HLA type information where available.

Table 1 provides a summary of the number of individuals and HLA alleles present in all reference and validation panels.

## Results

We have repeated some of the initial HapMap-based experiments from Dilthey et al. [1] to investigate the effects of the methodological innovations proposed in this paper (see Supporting Table S1 and Section “Properties of the presented model and parameter inference” in the Supporting Text S1). We find that allowing for path uncertainty has a positive effect across all examined loci. The additional localization criteria, though theoretically appealing, do not consistently improve accuracy across loci (see Supporting Text S1, Section “Properties of the presented model and parameter inference”). Based on our initial experiments, localization is not used for *HLA-B* and *HLA-DRB1* .

On a homogeneous reference panel (first experiment, GS), HLA*IMP:02 achieves the same level of performance as HLA*IMP (see Table 2). Measured at six classical HLA loci (*HLA-A* , *-B* , *-C* , *-DQA1* , *-DQB1* and *-DRB1*), HLA*IMP:02 achieves an average 4-digit resolution accuracy of 94% at an average call rate of 97%, vs. 93% accuracy at a call rate of 97% for HLA*IMP:01 (call threshold T = 0.7 for HLA*IMP:01 and matched to obtain equal or higher call rates for HLA*IMP:02). Locus-specific performance is very similar for both models. We observe the lowest accuracy at *HLA-DQA1* (88%) and the lowest call rate at *HLA-DRB1* (90%).

**Table 2:** Baseline validation on a homogeneous reference panel.

Threshold	Locus	# Validated	HLA*IMP:02			HLA:IMP:01		
			Call Rate	Accuracy	T	Call Rate	Accuracy	T
T = 0.00	*HLA-A*	574	1.00	0.96		1.00	0.90	
	*HLA-B*	2002	1.00	0.90		1.00	0.93	
	*HLA-C*	596	1.00	0.96		1.00	0.96	
	*HLA-DQA1*	446	1.00	0.87		1.00	0.87	
	*HLA-DQB1*	758	1.00	0.98		1.00	0.97	
	*HLA-DRB1*	1730	1.00	0.88		1.00	0.89	
T = Matched	*HLA-A*	574	0.96	0.96	0.55	0.94	0.91	0.700
	*HLA-B*	2002	0.98	0.92	0.40	0.98	0.94	0.700
	*HLA-C*	596	0.99	0.96	0.60	0.99	0.97	0.700
	*HLA-DQA1*	446	0.99	0.88	0.40	0.99	0.88	0.700
	*HLA-DQB1*	758	0.99	0.98	0.60	0.99	0.97	0.700
	*HLA-DRB1*	1730	0.90	0.93	0.60	0.90	0.93	0.700

Non-thresholded and thresholded HLARES validation results for HLA*IMP:02 and HLA*IMP:01: the complete GS is used to impute HLARES_EU samples. Accuracy (PPV) is measured at 4-digit resolution. “# Validated” refers to the number of validated alleleles (pre-thresholding). Note that the call threshold for HLA*IMP:02 was matched to obtain equal or higher call rates than with HLA*IMP:01.

On a heterogeneous reference panel (second experiment, GS&HLARES_EU 2/3), HLA*IMP:02 achieves an average accuracy of 97% at an average call rate of 97% (see Table 3). HLA*IMP:01, in contrast, achieves an average accuracy of 93% at an average call rate of 93% (using a call threshold of T = 0.7 for both models). The most problematic locus for HLA*IMP:02 is *HLA-DRB1* , with an achieved accuracy/call rate of 95%/91%. Even without call threshold, HLA*IMP:02 achieves an all-loci average accuracy of 96% (vs. 90% for HLA*IMP:01). At T = 0.00, HLA*IMP:02 outperforms HLA*IMP:01 at every locus, by 6% on average. Applied to *HLA-DPB1* and the allelic state of the *DRB* paralogs (see Supporting Table S3), HLA*IMP:02 achieves an accuracy of 90% on *DPB1* without any call threshold. Due to the limitations of the data set, we can only evaluate the performance at the *DRB* paralogous loci at 2-digit resolution, including one pseudo-allele for absence from a haplotype. We find the imputations to be correct in 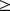
94% of cases (T = 0.00, very similar results obtained for HLA*IMP:01, data not shown). HLA*IMP:02 produces well-calibrated imputations (see Supporting Figure S1).

**Table 3:** Multi-population European validation results.

Threshold	Locus	# Validated	HLA*IMP:02		HLA:IMP:01	
			Call Rate	Accuracy	Call Rate	Accuracy
T = 0.00	*HLA-A*	808	1.00	0.97	1.00	0.91
	*HLA-B*	1646	1.00	0.95	1.00	0.89
	*HLA-C*	752	1.00	0.96	1.00	0.91
	*HLA-DQA1*	194	1.00	0.97	1.00	0.87
	*HLA-DQB1*	934	1.00	0.98	1.00	0.92
	*HLA-DRB1*	1358	1.00	0.91	1.00	0.87
T = 0.70	*HLA-A*	808	0.98	0.97	0.94	0.94
	*HLA-B*	1646	0.96	0.97	0.93	0.92
	*HLA-C*	752	0.99	0.97	0.94	0.94
	*HLA-DQA1*	194	0.96	0.98	0.93	0.90
	*HLA-DQB1*	934	0.99	0.98	0.94	0.94
	*HLA-DRB1*	1358	0.91	0.95	0.89	0.92

Medium heterogeneity non-thresholded and thresholded cross-validation results for HLA*IMP:02 and HLA*IMP:01: GS&HLARES_EU 2/3 is used to impute GS&HLARES_EU 1/3. Accuracy (PPV) is measured at 4-digit resolution. “# Validated” refers to the number of validated alleleles (pre-thresholding).

**Figure 3:**
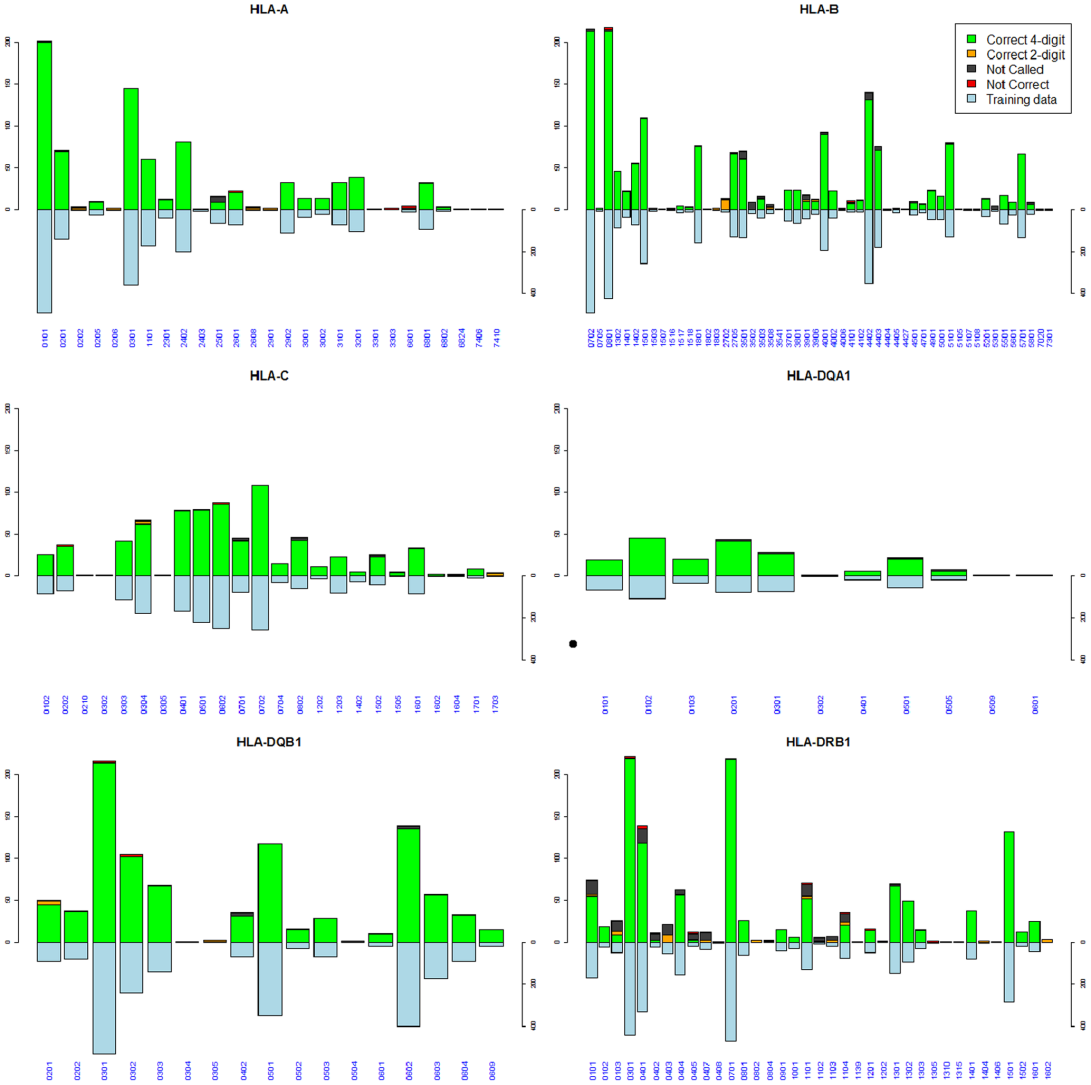
Per-allele accuracies on a diverse European reference panel (HLA*IMP:02). Per-allele analysis of HLA*IMP:02 imputation accuracy for six classical loci in the GS&HLARES_EU cross-European validation experiment at a call threshold of T = 0.70. The x-axis represents the different HLA alleles in the validation panel. The downward blue bars indicate how often each allele appears in the reference panel (the GS&HLARES_EU 2/3 dataset). Imputation success is indicated by the upward stack plots: green indicates correct imputations at 4-digit HLA type resolution; orange indicates correct imputations at 2-digit resolution; black indicates alleles below the call threshold; red indicates incorrect imputations. Non-calls and imputations which are only correct at 2-digit resolution accumulate in the alleles which are rare or not present at all in the reference panel.

By analyzing allele- and locus-specific error profiles, we can identify factors influencing the imputation accuracy of HLA*IMP:02 (see Figure 3). First, we note that most alleles are imputed reliably at 4-digit resolution, in particular those with higher frequencies in the reference panel. Alleles that exhibit problems at 4-digit imputation are typically correctly imputed at 2-digit resolution. Second, we can distinguish between at least three classes of problems. Some alleles, for example HLA-A*33:01, are not present in the reference dataset at all. They can therefore not be correctly imputed. Other alleles, for example HLA-B*27:02, are present in the reference dataset, but at low frequencies. Non-calls and 4-digit errors accumulate for these alleles. Third, some alleles, for example DRB1*01:01, are better represented in the reference panel, but there are still some problems with imputing them correctly. We note that these error modes are also seen in HLA*IMP:01 and that the identified classes of error also apply to the homogeneous reference experiment (see Supporting Figures S2, S3, S4). Finally, there is another abundant type of error, seen only in HLA*IMP:01 and not observed in the low heterogeneity case, which drives the observed drop in performance difference relative to HLA*IMP:02: classification problems for well-represented alleles. It seems likely that this is due to within-Europe population structure and heterogeneity in haplotype backgrounds, which the model of HLA*IMP:01 cannot take into account appropriately. We provide allele-specific measures of sensitivity, specificity, PPV and 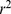, based on HLA*IMP:02, for the first two experiments in Supporting Tables S4 and S5.

To investigate how strong an effect the utilized SNP genotyping array and missing data in the imputation dataset will have on expected accuracy, we carry out a variation of the second experiment. Instead of separately evaluating a range of genotyping platforms and missingness profiles, we present two generic experiments, focusing on SNP density in the xMHC region: we randomly delete 70% and 90% of the SNP genotypes from the inference panel (independently for each individual, to minimize sampling effects), while the graph from the second experiment remains unchanged. In the 70% scenario, each individual remains with 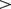
1500 SNPs in the xMHC region, which is comparable to the SNP density of many 500 K arrays. In the 90% scenario, approximately 600 SNPs in the xMHC region remain, which is substantially less than the number provided by older 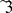
00 K genotyping arrays. We observe that even with low SNP densities, the observed performance of HLA*IMP:02 is relatively stable: Setting 70% of the SNP genotypes in the inference panel (GS&HLARES_EU 1/3) to “missing”, the drop in achieved per-locus accuracy is 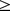
1% (at a call threshold of T = 0.00, see Table 4). Setting 90% of the SNP genotypes in the inference panel to “missing”, the maximum loss in accuracy is 5% for all loci but *DQA1* (probably related to the smaller amount of reference data for this locus, see Table 1), where it is 7%. Of note, many reference panel SNPs are present on the Immunochip platform; repeating the second experiment constrained to the Immunochip SNP set for the imputation panel shows virtually the same results as the 70% experiment (data not shown).

**Table 4:** Missing data in the inference panel.

Locus	# Validated	70% missing	90% missing
*HLA-A*	808	0.96	0.94
*HLA-B*	1646	0.95	0.93
*HLA-C*	752	0.95	0.94
*HLA-DQA1*	194	0.96	0.90
*HLA-DQB1*	934	0.97	0.95
*HLA-DRB1*	1358	0.90	0.86

4-digit resolution accuracies (PPV) when 70% and 90% of the inference panel SNP genotypes (GS&HLARES_EU 1/3) in the second experiment are randomly set to “missing”. No call threshold is employed. “# Validated” refers to the number of validated alleleles.

Increasing the heterogeneity in the reference panel (third experiment, GS&HLARES_ALL 2/3) by including individuals of other ethnicities (African-ancestry, Asian, Hispanic, third experiment) only slightly decreases the achieved performance on the European validation samples for HLA*IMP:02 (see Table 5), yielding an average accuracy of 97% and an average call rate of 95% (T = 0.70). At 4-digit resolution, performance on the non-European samples is markedly lower, with an average accuracy and an average call rate of 87% (T = 0.70). Imputation accuracy is lowest for the Asian samples (average accuracy 76% at T = 0.00) and comparable for African-ancestry and Hispanic samples (84% and 85% respectively, at T = 0.00). There are more pronounced locus-specific differences in the non-European validation data: In the African-ancestry samples and at T = 0.00, for example, accuracy at *HLA-DRB1* is at 71%, whereas it is at 97% at *HLA-C* . At 2-digit resolution, alleles are imputed more reliably: average accuracy at T = 0.00 is 90% for Asian samples (ranging from 78% at *HLA-B* to 98% at *HLA-DRB1*); 93% for samples of African ancestry (ranging from 82% at *HLA-B* to 100% at *HLA-C/DQA1/DQB1*); and 99% for Hispanic samples (ranging from 97% at *HLA-B* to 100% at all other loci);

**Table 5:** Multi-ethnic validation results.

Threshold	Population	Locus	# Validated	Call Rate	Accuracy 4-digit	Accuracy 2-digit
T = 0.00	African-American/African	*HLA-A*	30	1.00	0.73	0.83
		*HLA-B*	44	1.00	0.73	0.82
		*HLA-C*	30	1.00	0.97	1.00
		*HLA-DQA1*	28	1.00	1.00	1.00
		*HLA-DQB1*	30	1.00	0.87	1.00
		*HLA-DRB1*	34	1.00	0.71	0.91
	Asian	*HLA-A*	28	1.00	0.79	0.96
		*HLA-B*	110	1.00	0.68	0.78
		*HLA-C*	28	1.00	0.82	0.89
		*HLA-DQA1*	22	1.00	0.73	0.91
		*HLA-DQB1*	36	1.00	0.83	0.89
		*HLA-DRB1*	102	1.00	0.72	0.98
	European	*HLA-A*	824	1.00	0.96	0.97
		*HLA-B*	1662	1.00	0.95	0.98
		*HLA-C*	752	1.00	0.97	0.99
		*HLA-DQA1*	206	1.00	0.96	0.99
		*HLA-DQB1*	924	1.00	0.97	0.99
		*HLA-DRB1*	1356	1.00	0.90	0.99
	Hispanic	*HLA-A*	28	1.00	0.82	1.00
		*HLA-B*	126	1.00	0.63	0.97
		*HLA-C*	36	1.00	0.92	1.00
		*HLA-DQA1*	28	1.00	0.93	1.00
		*HLA-DQB1*	40	1.00	0.97	1.00
		*HLA-DRB1*	128	1.00	0.80	0.98
T = 0.70	African-American/African	*HLA-A*	30	0.93	0.79	0.89
		*HLA-B*	44	0.89	0.79	0.85
		*HLA-C*	30	1.00	0.97	1.00
		*HLA-DQA1*	28	1.00	1.00	1.00
		*HLA-DQB1*	30	0.93	0.89	1.00
		*HLA-DRB1*	34	0.59	1.00	1.00
	Asian	*HLA-A*	28	0.96	0.81	1.00
		*HLA-B*	110	0.71	0.85	0.91
		*HLA-C*	28	0.86	0.79	0.88
		*HLA-DQA1*	22	0.82	0.78	0.94
		*HLA-DQB1*	36	0.83	0.90	0.93
		*HLA-DRB1*	102	0.74	0.83	1.00
	European	*HLA-A*	824	0.95	0.97	0.98
		*HLA-B*	1662	0.95	0.97	0.99
		*HLA-C*	752	0.99	0.97	0.99
		*HLA-DQA1*	206	0.97	0.97	0.99
		*HLA-DQB1*	924	0.99	0.98	0.99
		*HLA-DRB1*	1356	0.87	0.95	0.99
	Hispanic	*HLA-A*	28	1.00	0.82	1.00
		*HLA-B*	126	0.75	0.73	0.97
		*HLA-C*	36	0.94	0.97	1.00
		*HLA-DQA1*	28	0.86	0.96	1.00
		*HLA-DQB1*	40	0.95	1.00	1.00
		*HLA-DRB1*	128	0.73	0.88	1.00

High heterogeneity non-thresholded and thresholded cross-validation results for HLA*IMP:02, stratified by ethnicity of the imputed samples. GS&HLARES_ALL 2/3 is used to impute GS&HLARES_ALL 1/3. Accuracy (PPV) is measured at 4-digit resolution and at 2-digit resolution. “# Validated” refers to the number of validated alleleles (pre-thresholding).

## Discussion

Better imputation of classical HLA alleles is an important goal in enabling association studies to understand the genetic risk of many complex and infectious diseases. We have developed HLA*IMP:02, a statistical model for the imputation of classical HLA types, which attempts to address problems arising in performing imputation from multiple heterogeneous (both in experimental origin and ethnicity) data sets. We have shown that HLA*IMP:01 (our previous method; [1], [2]) and HLA*IMP:02 achieve similar levels of performance on homogeneous reference panels, but that HLA*IMP:02 clearly outperforms HLA*IMP:01 on heterogeneous European reference panels, yielding accuracies and call rates 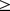
95% at 4-digit resolution in nearly all European scenarios. Using HLA*IMP:02 instead of HLA*IMP:01 can therefore be expected to increase power and accuracy in cross-European genome-wide association studies.

The improved performance of HLA*IMP:02 (when compared with HLA*IMP:01) is likely due to the path-based approach that allows for HLA alleles to appear on multiple haplotype backgrounds, a known consequence of population stratification in the HLA region. To further investigate this hypothesis, we have examined the local haplotype structure around the HLA-A*02:01 allele in GS&HLARES_EU, as inferred (and used) by HLA*IMP:01 (Supporting Figure S5, part B). From visual inspection of the figure, it is clear that there are at least three major haplotypic backgrounds for 02:01 (when inspecting the corresponding figure for the GS, we find two major haplotypic backgrounds; Supporting Figure S5, part A). What is more, when comparing the haplotypes that HLA*IMP:01 correctly imputes with those that it doesn't, we find that there are features which appear virtually exclusively in the second group (marked in S11 part B). Interestingly, these features are also present in the group of haplotypes that serve as reference panel, but the model does not seem to utilize this information in the right way. This is consistent with our interpretation that the model of HLA*IMP:01 does not cope well with haplotypic heterogeneity. HLA*IMP:02, on the other hand, can accommodate haplotypic heterogeneity and imputes A*02:01 nearly perfectly in the same experiment.

The observed performance of HLA*IMP:02 is relatively stable under high levels of missing data in the inference panel. This property represents an important improvement upon HLA*IMP:01, which offered no conceptually consistent way (except for repeating the computationally intensive process of SNP selection) towards dealing with missing SNPs in the inference panel. Of note, the HLA*IMP:02 back end web service will automatically carry out the SNP density experiment presented here, constraining the set of available SNPs to those found in the user dataset. The results from this experiment (including average per-locus accuracies and PPV, sensitivity and specificity for each allele) are included in the archive file which contains the main imputations.

The model of HLA*IMP:02 could handle pre-phased data in a straightforward way. There is no evidence to suggest that recent encouraging results from SNP genotype imputation [23] do not apply to pre-phasing with the aim of HLA type imputation. However, in light of the complex regional haplotype structure and high levels of diversity, we believe that the effect of pre-phasing on HLA type imputation accuracy needs to be studied in more detail.

**Figure 4:**
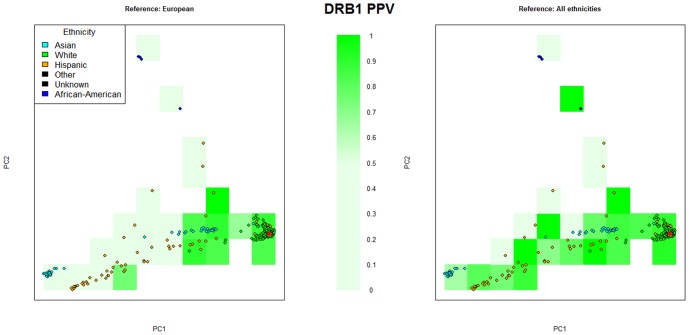
Accuracy comparison between complete and European-restricted reference panels. PCA-stratified accuracy comparison (*HLA-DRB1*) between the complete reference panel (GS&HLARES_ALL, right plot) and a European-restricted reference panel (left side) for the high heterogeneity scenario (imputing GS&HLARES_ALL 1/3, only samples from HLARES displayed). In each quadrant, mean accuracy (PPV) is indicated by color. The red triangle indicates the (approximate) centre of the European reference data. Note that incorporating the non-European reference data increases accuracy in particular for the non-European samples.

At 2-digit resolution, HLA*IMP:02 achieves average accuracies 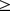
90% for all tested ethnicities using a multi-ethnic reference panel. These results suggest that the model's ability to deal with heterogeneity in the reference set extends to highly diverse panels. Moreover, extensions of the reference panel in a way that matches imputation study panels can be expected to furthermore increase (4-digit) performance, in particular for samples that are not well-represented by the current reference. We illustrate this effect in Figure 4 for *HLA-DRB1* , one of the more challenging loci for HLA type imputation. The figure displays samples from HLARES_ALL 1/3 stratified by the samples' first two principal components (it is well-known that PCA can be used to control for population stratification [24] and is informative of relatedness [25]). In one experiment, we use an exclusively European reference to impute the samples (left-hand panel). In the other experiment, we make use of the full reference panel GS&HLARES ALL 2/3 (right-hand panel). Particularly samples in the periphery of PC space benefit from improving reference panel size and match with the imputation panel, whereas samples in the proximity of European data are hardly affected. Averaged over all loci, accuracy for the non-European samples increases by 8% when including the non-European reference data (data not shown). These observations are consistent with results from SNP genotype imputation, where using matched and diverse reference panels is also known to have a positive effect on accuracy [11], [12], [26], [27].

In summary, the model of HLA*IMP:02 contributes to solving the important challenge of making HLA type inference from combined multi-population reference panels. Raising the accuracy of 4-digit imputation accuracy for non-European populations to the level currently observed for European samples is an important future goal that will require collection of reference data from other populations. However, the framework developed here should enable such integration to happen without compromising accuracy in European-ancestry populations.

## Supporting Information

Figure S1 Calibration HLA*IMP:02. Calibration plot HLA*IMP:02, second experiment, medium heterogeneity. The red points show expected (x-axis) and achieved mean accuracies (y-axis) in each bin of step size 0.1, and the blue line is a plot of x = y. Note that the first four data points (bins 0–3) are only based on 37 individuals.
(TIF)

Figure S2 Per-allele analysis for HLA*IMP:02/HLARES. Per-allele analysis of HLA*IMP:02 imputation accuracy for six classical loci in the HLARES validation experiment (first experiment, homogeneous reference) at a call threshold of T = 0.70. The x-axis represents the different HLA alleles in the validation panel. The downward blue bars indicate how often each allele appears in the reference panel (the GS dataset). Imputation success is indicated by the upward stack plots: green indicates correct imputations at 4-digit HLA type resolution; orange indicates correct imputations at 2-digit resolution; black indicates alleles below the call threshold; red indicates incorrect imputations.
(TIF)

Figure S3 Per-allele analysis for HLA*IMP:01/HLARES. Per-allele analysis of HLA*IMP:01 imputation accuracy for six classical loci in the HLARES validation experiment (first experiment, homogeneous reference) at a call threshold of T = 0.70. The x-axis represents the different HLA alleles in the validation panel. The downward blue bars indicate how often each allele appears in the reference panel (the GS dataset). Imputation success is indicated by the upward stack plots: green indicates correct imputations at 4-digit HLA type resolution; orange indicates correct imputations at 2-digit resolution; black indicates alleles below the call threshold; red indicates incorrect imputations.
(TIF)

Figure S4 Per-allele analysis for HLA*IMP:01/GS&HLARES_EU. Per-allele analysis of HLA*IMP:01 imputation accuracy for six classical loci in the GS&HLARES_EU validation experiment (second experiment, medium heterogeneity reference) at a call threshold of T = 0.70. The x-axis represents the different HLA alleles in the validation panel. The downward blue bars indicate how often each allele appears in the reference panel (the GS dataset). Imputation success is indicated by the upward stack plots: green indicates correct imputations at 4-digit HLA type resolution; orange indicates correct imputations at 2-digit resolution; black indicates alleles below the call threshold; red indicates incorrect imputations.
(TIF)

Figure S5 Barcode plot for HLA-A*02:01 in in GS and GS&HLARES_EU. This plot shows the inferred haplotype structure (“barcode plot”) for HLA-A*02:01 in the first (based on GS, part A) and second experiment (based on GS&HLARES_EU, part B). Each row represents one haplotype, and each SNP is depicted as a little square. The colouring of the boxes indicates whether the haplotype carries the major SNP allele (bright box) or a minor allele (dark box). The black/white rows represent the haplotypes carrying the 02:01 allele in the reference panel. Red and green rows represent haplotypes carrying 02:01 in the validation panel, with green indicating successful imputation and red indicating misimputation. We only show SNPs selected by HLA*IMP:01 in the process of SNP selection, and the inferred haplotypes are taken from the phased reference panel for HLA*IMP:01. The inferred haplotype structure in the second experiment in more complex than in the first experiment. Comparing correctly and incorrectly imputed haplotypes in the second experiment, it is clear that there are features (highlighted) which appear virtually exclusively in incorrectly imputed haplotypes (although they are present in the reference panel). Note that A*02:01 is imputed virtually perfectly by HLA*IMP:02 in this experiment, consistent with our hypothesis that HLA*IMP:02 is more tolerant of heterogeneous haplotype structures.
(TIF)

Table S1
**HapMap-based BC58 validation accuracy.** Accuracies (PPV) for the HapMap-based BC58 validation, as described in Leslie et al. [2] and Dilthey et al. [1]. No call threshold is employed. The column “HLA*IMP:02” refers to the full model with error parameters ! =  0 and localization (other parameters set to accommodate the much reduced sample size). In column I, the error probabilities for sampling from the graph (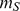) and for building the graph 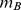 are set to 0 (all other parameters equal to the column “HLA*IMP:02”). In column II, the error probability for building the graph is set to 0, and in column III, the error probability for sampling from the graph is set to 0. In column IV, localization is deactivated.(DOCX)

Table S2 **Countries and ethnicities in HLARES.** Country and ethnicity of samples in the HLARES_EU and HLARES_ALL datasets.
(DOCX)

Table S3 **HLA-DPB1 and DRB3-5.** HLARES_EU cross validation for additional loci and structural variation (second experiment, medium heterogeneity): 2/3 of the HLARES_EU dataset are used as reference to impute the remaining 1/3. No call threshold is employed. Accuracy (PPV) for HLA-DPB1 measured at 4-digit resolution, at 2-digit resolution (including one pseudo-allele for absence) for DRB orthologs.
(DOCX)

Table S4
**HLA-DPB1 and DRB3-5.** Allele-specific sensitivity, specificity, PPV and 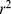 for the first experiment (HLA*IMP:02, GS 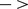 HLARES_EU). “NValidation” specifies how often an allele appears in the validation data (according to classical typing results, which we treat as the truth in this experiment). “NImputation” specifies how often an allele appears in the imputations for the validation data. The following columns specify sensitivity, specificity, PPV and r2 for each allele. All numbers are based on “best-guess” called alleles.(DOCX)

Table S5
**HLA-DPB1 and DRB3-5.** Allele-specific sensitivity, specificity, PPV and 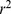 for the second experiment (HLA*IMP:02, GS&HLARES_EU 2/3 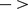 GS&HLARES_EU 1/3). “NValidation” specifies how often an allele appears in the validation data (according to classical typing results, which we treat as the truth in this experiment). “NImputation” specifies how often an allele appears in the imputations for the validation data. The following columns specify sensitivity, specificity, PPV and r2 for each allele. All numbers are based on “best-guess” called alleles.(DOCX)

Text S1 The HLA*IMP:02 model and algorithms. Mathematical and algorithmic characterization of the haplotype graph model of HLA*IMP:02, allowing for integrating over path uncertainty and localization.
(PDF)
