# Supplementary figures and images for: Multi-Population Classical HLA Type Imputation
Source: PLoS Comput Biol. 2013 Feb 14;9(2):e1002877. doi: 10.1371/journal.pcbi.1002877 (PMC3572961; doi:10.1371/journal.pcbi.1002877)

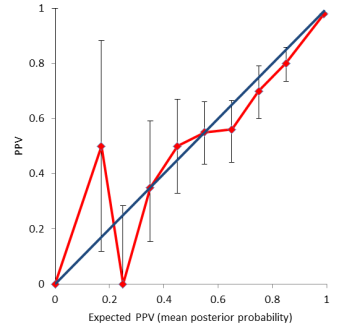

Supplement: Figure S1 — Calibration HLA*IMP:02. Calibration plot HLA*IMP:02, second experiment, medium heterogeneity. The red points show expected (x-axis) and achieved mean accuracies (y-axis) in each bin of step size 0.1, and the blue line is a plot of x = y. Note that the first four data points (bins 0–3) are only based on 37 individuals. (TIF) [file pcbi.1002877.s001.tif]
