# Supplementary material for: Multi-Population Classical HLA Type Imputation
Source: PLoS Comput Biol. 2013 Feb 14;9(2):e1002877. doi: 10.1371/journal.pcbi.1002877 (PMC3572961; doi:10.1371/journal.pcbi.1002877)
Supplement: Table S1 — HapMap-based BC58 validation accuracy. Accuracies (PPV) for the HapMap-based BC58 validation, as described in Leslie et al. [2] and Dilthey et al. [1]. No call threshold is employed. The column “HLA*IMP:02” refers to the full model with error parameters ! = 0 and localization (other parameters set to accommodate the much reduced sample size). In column I, the error probabilities for sampling from the graph () and for building the graph are set to 0 (all other parameters equal to the column “HLA*IMP:02”). In column II, the error probability for building the graph is set to 0, and in column III, the error probability for sampling from the graph is set to 0. In column IV, localization is deactivated. (DOCX) [file pcbi.1002877.s006.docx]

## Supplementary Table S1

| **Accuracy** | **HLA*IMP:2** | **I** | **II** | **III** | **IV** |
| --- | --- | --- | --- | --- | --- |
| HLA-A | 0.92 | 0.82 | 0.74 | 0.89 | 0.90 |
| HLA-B | 0.78 | 0.56 | 0.55 | 0.66 | 0.83 |
| HLA-DQB1 | 0.84 | 0.58 | 0.58 | 0.72 | 0.83 |
| HLA-DRB1 | 0.70 | 0.50 | 0.49 | 0.68 | 0.74 |
| Accuracies (PPV) for the HapMap-based BC58 validation, as described in [Leslie et al. (2008](#_ENREF_2)) and [Dilthey et al. (2011](#_ENREF_1)). No call threshold is employed. The column “HLA*IMP:02” refers to the full model with error parameters != 0 and localization. In column I, the error probabilities for sampling from the graph (*m_S_*) and for building the graph *m_B_* are set to 0 (all other parameters equal to the full model). In column II, the error probability for building the graph is set to 0, and in column III, the error probability for sampling from the graph is set to 0. In column IV, localization is deactivated. | | | | | |
