# Supplementary material for: Multi-Population Classical HLA Type Imputation
Source: PLoS Comput Biol. 2013 Feb 14;9(2):e1002877. doi: 10.1371/journal.pcbi.1002877 (PMC3572961; doi:10.1371/journal.pcbi.1002877)
Supplement: Table S2 — Countries and ethnicities in HLARES. Country and ethnicity of samples in the HLARES_EU and HLARES_ALL datasets. (DOCX) [file pcbi.1002877.s007.docx]

## Supplementary Table S2

Country and ethnicity of samples in the HLARES_EU and HLARES_ALL datasets.
