# Supplementary material for: Multi-Population Classical HLA Type Imputation
Source: PLoS Comput Biol. 2013 Feb 14;9(2):e1002877. doi: 10.1371/journal.pcbi.1002877 (PMC3572961; doi:10.1371/journal.pcbi.1002877)
Supplement: Table S3 — HLA-DPB1 and DRB3-5. HLARES_EU cross validation for additional loci and structural variation (second experiment, medium heterogeneity): 2/3 of the HLARES_EU dataset are used as reference to impute the remaining 1/3. No call threshold is employed. Accuracy (PPV) for HLA-DPB1 measured at 4-digit resolution, at 2-digit resolution (including one pseudo-allele for absence) for DRB orthologs. (DOCX) [file pcbi.1002877.s008.docx]

## Supplementary Table S3

| **Locus** | **# Validated Alleles** | **PPV (Accuracy)** |  |  |
| --- | --- | --- | --- | --- |
| *HLA-DPB1* | 48 | 0.90 |  |  |
| *HLA-DRB3* | 190 | 0.94 |  |  |
| *HLA-DRB4* | 190 | 0.98 |  |  |
| *HLA-DRB5* | 190 | 0.99 |  |  |
|  |  |  |  |  |
| HLARES_EU cross validation for additional loci and structural variation (second experiment, medium heterogeneity): 2/3 of the HLARES_EU dataset are used as reference to impute the remaining 1/3. No call threshold is employed. Accuracy (PPV) for *HLA-DPB1* measured at 4-digit resolution, at 2-digit resolution (including one pseudo-allele for absence) for *DRB* orthologs. | | | | |
