# Supplementary material for: Multi-Population Classical HLA Type Imputation
Source: PLoS Comput Biol. 2013 Feb 14;9(2):e1002877. doi: 10.1371/journal.pcbi.1002877 (PMC3572961; doi:10.1371/journal.pcbi.1002877)
Supplement: Table S4 — HLA-DPB1 and DRB3-5. Allele-specific sensitivity, specificity, PPV and for the first experiment (HLA*IMP:02, GS HLARES_EU). “NValidation” specifies how often an allele appears in the validation data (according to classical typing results, which we treat as the truth in this experiment). “NImputation” specifies how often an allele appears in the imputations for the validation data. The following columns specify sensitivity, specificity, PPV and r2 for each allele. All numbers are based on “best-guess” called alleles. (DOCX) [file pcbi.1002877.s009.docx]

## Supplementary Table S4

| **Locus** | **Allele** | **NValidation** | **NImputation** | **Sensitivity** | **Specificity** | **PPV** | **r^2^** |
| --- | --- | --- | --- | --- | --- | --- | --- |
| A | 0101 | 92 | 93 | 1.00 | 1.00 | 0.99 | 0.99 |
| A | 0102 | 1 | 0 | 0.00 | 1.00 | NA | 0.00 |
| A | 0201 | 157 | 158 | 1.00 | 1.00 | 0.99 | 0.99 |
| A | 0202 | 2 | 0 | 0.00 | 1.00 | NA | 0.00 |
| A | 0205 | 4 | 6 | 1.00 | 1.00 | 0.67 | 0.66 |
| A | 0206 | 1 | 0 | 0.00 | 1.00 | NA | 0.00 |
| A | 0301 | 72 | 73 | 1.00 | 1.00 | 0.99 | 0.98 |
| A | 0317 | 1 | 0 | 0.00 | 1.00 | NA | 0.00 |
| A | 1101 | 38 | 39 | 1.00 | 1.00 | 0.97 | 0.97 |
| A | 1102 | 1 | 0 | 0.00 | 1.00 | NA | 0.00 |
| A | 2301 | 13 | 13 | 1.00 | 1.00 | 1.00 | 1.00 |
| A | 2402 | 48 | 49 | 1.00 | 1.00 | 0.98 | 0.98 |
| A | 2403 | 1 | 0 | 0.00 | 1.00 | NA | 0.00 |
| A | 2501 | 17 | 16 | 0.82 | 1.00 | 0.88 | 0.73 |
| A | 2601 | 25 | 31 | 0.92 | 0.99 | 0.74 | 0.66 |
| A | 2901 | 4 | 0 | 0.00 | 1.00 | NA | 0.00 |
| A | 2902 | 21 | 25 | 1.00 | 0.99 | 0.84 | 0.83 |
| A | 3001 | 9 | 9 | 1.00 | 1.00 | 1.00 | 1.00 |
| A | 3002 | 7 | 7 | 1.00 | 1.00 | 1.00 | 1.00 |
| A | 3101 | 12 | 14 | 1.00 | 1.00 | 0.86 | 0.85 |
| A | 3201 | 27 | 27 | 1.00 | 1.00 | 1.00 | 1.00 |
| A | 3301 | 1 | 0 | 0.00 | 1.00 | NA | 0.00 |
| A | 3303 | 1 | 0 | 0.00 | 1.00 | NA | 0.00 |
| A | 6601 | 5 | 0 | 0.00 | 1.00 | NA | 0.00 |
| A | 6801 | 12 | 14 | 1.00 | 1.00 | 0.86 | 0.85 |
| A | 6802 | 1 | 0 | 0.00 | 1.00 | NA | 0.00 |
| A | 6824 | 1 | 0 | 0.00 | 1.00 | NA | 0.00 |
|  |  |  |  |  |  |  |  |
| B | 0702 | 240 | 242 | 0.99 | 1.00 | 0.98 | 0.96 |
| B | 0704 | 1 | 0 | 0.00 | 1.00 | NA | 0.00 |
| B | 0705 | 9 | 0 | 0.00 | 1.00 | NA | 0.00 |
| B | 0801 | 199 | 201 | 1.00 | 1.00 | 0.99 | 0.99 |
| B | 1302 | 74 | 76 | 1.00 | 1.00 | 0.97 | 0.97 |
| B | 1401 | 14 | 12 | 0.86 | 1.00 | 1.00 | 0.86 |
| B | 1402 | 44 | 46 | 1.00 | 1.00 | 0.96 | 0.96 |
| B | 1501 | 132 | 139 | 0.98 | 0.99 | 0.93 | 0.90 |
| B | 1502 | 1 | 0 | 0.00 | 1.00 | NA | 0.00 |
| B | 1503 | 1 | 4 | 1.00 | 1.00 | 0.25 | 0.25 |
| B | 1510 | 1 | 0 | 0.00 | 1.00 | NA | 0.00 |
| B | 1516 | 1 | 0 | 0.00 | 1.00 | NA | 0.00 |
| B | 1517 | 12 | 8 | 0.67 | 1.00 | 1.00 | 0.66 |
| B | 1518 | 1 | 0 | 0.00 | 1.00 | NA | 0.00 |
| B | 1801 | 130 | 128 | 0.93 | 1.00 | 0.95 | 0.86 |
| B | 1802 | 1 | 0 | 0.00 | 1.00 | NA | 0.00 |
| B | 1803 | 5 | 0 | 0.00 | 1.00 | NA | 0.00 |
| B | 2702 | 21 | 0 | 0.00 | 1.00 | NA | 0.00 |
| B | 2705 | 66 | 89 | 1.00 | 0.99 | 0.74 | 0.73 |
| B | 3501 | 93 | 131 | 0.92 | 0.98 | 0.66 | 0.59 |
| B | 3502 | 17 | 0 | 0.00 | 1.00 | NA | 0.00 |
| B | 3503 | 37 | 27 | 0.65 | 1.00 | 0.89 | 0.57 |
| B | 3508 | 13 | 2 | 0.08 | 1.00 | 0.50 | 0.04 |
| B | 3517 | 1 | 0 | 0.00 | 1.00 | NA | 0.00 |
| B | 3541 | 1 | 0 | 0.00 | 1.00 | NA | 0.00 |
| B | 3555 | 1 | 0 | 0.00 | 1.00 | NA | 0.00 |
| B | 3701 | 27 | 27 | 0.96 | 1.00 | 0.96 | 0.93 |
| B | 3801 | 57 | 71 | 0.95 | 0.99 | 0.76 | 0.72 |
| B | 3901 | 31 | 19 | 0.52 | 1.00 | 0.84 | 0.46 |
| B | 3906 | 7 | 16 | 0.86 | 0.99 | 0.38 | 0.32 |
| B | 3910 | 1 | 0 | 0.00 | 1.00 | NA | 0.00 |
| B | 3924 | 2 | 0 | 0.00 | 1.00 | NA | 0.00 |
| B | 4001 | 84 | 84 | 0.99 | 1.00 | 0.99 | 0.97 |
| B | 4002 | 42 | 44 | 0.95 | 1.00 | 0.91 | 0.83 |
| B | 4006 | 4 | 0 | 0.00 | 1.00 | NA | 0.00 |
| B | 4101 | 10 | 11 | 1.00 | 1.00 | 0.91 | 0.91 |
| B | 4102 | 12 | 12 | 1.00 | 1.00 | 1.00 | 1.00 |
| B | 4201 | 1 | 0 | 0.00 | 1.00 | NA | 0.00 |
| B | 4402 | 157 | 172 | 0.99 | 0.99 | 0.90 | 0.88 |
| B | 4403 | 100 | 112 | 0.99 | 0.99 | 0.88 | 0.86 |
| B | 4405 | 12 | 1 | 0.00 | 1.00 | 0.00 | 0.00 |
| B | 4427 | 1 | 0 | 0.00 | 1.00 | NA | 0.00 |
| B | 4501 | 10 | 8 | 0.80 | 1.00 | 1.00 | 0.80 |
| B | 4701 | 5 | 5 | 1.00 | 1.00 | 1.00 | 1.00 |
| B | 4901 | 34 | 34 | 0.94 | 1.00 | 0.94 | 0.88 |
| B | 5001 | 31 | 31 | 0.97 | 1.00 | 0.97 | 0.93 |
| B | 5101 | 97 | 104 | 0.98 | 1.00 | 0.91 | 0.89 |
| B | 5105 | 1 | 0 | 0.00 | 1.00 | NA | 0.00 |
| B | 5107 | 2 | 0 | 0.00 | 1.00 | NA | 0.00 |
| B | 5108 | 2 | 0 | 0.00 | 1.00 | NA | 0.00 |
| B | 5201 | 24 | 22 | 0.92 | 1.00 | 1.00 | 0.92 |
| B | 5301 | 6 | 0 | 0.00 | 1.00 | NA | 0.00 |
| B | 5401 | 1 | 0 | 0.00 | 1.00 | NA | 0.00 |
| B | 5501 | 28 | 26 | 0.93 | 1.00 | 1.00 | 0.93 |
| B | 5601 | 21 | 26 | 1.00 | 1.00 | 0.81 | 0.80 |
| B | 5701 | 57 | 64 | 1.00 | 1.00 | 0.89 | 0.89 |
| B | 5703 | 1 | 0 | 0.00 | 1.00 | NA | 0.00 |
| B | 5801 | 14 | 8 | 0.57 | 1.00 | 1.00 | 0.57 |
| B | 7020 | 2 | 0 | 0.00 | 1.00 | NA | 0.00 |
| B | 7301 | 2 | 0 | 0.00 | 1.00 | NA | 0.00 |
|  |  |  |  |  |  |  |  |
| C | 0102 | 19 | 19 | 1.00 | 1.00 | 1.00 | 1.00 |
| C | 0202 | 31 | 33 | 1.00 | 1.00 | 0.94 | 0.93 |
| C | 0210 | 1 | 0 | 0.00 | 1.00 | NA | 0.00 |
| C | 0303 | 25 | 28 | 1.00 | 0.99 | 0.89 | 0.88 |
| C | 0304 | 38 | 37 | 0.92 | 1.00 | 0.95 | 0.86 |
| C | 0305 | 1 | 0 | 0.00 | 1.00 | NA | 0.00 |
| C | 0310 | 1 | 0 | 0.00 | 1.00 | NA | 0.00 |
| C | 0401 | 58 | 59 | 1.00 | 1.00 | 0.98 | 0.98 |
| C | 0409 | 1 | 0 | 0.00 | 1.00 | NA | 0.00 |
| C | 0501 | 46 | 45 | 0.98 | 1.00 | 1.00 | 0.98 |
| C | 0602 | 68 | 68 | 1.00 | 1.00 | 1.00 | 1.00 |
| C | 0701 | 88 | 85 | 0.97 | 1.00 | 1.00 | 0.96 |
| C | 0702 | 81 | 82 | 1.00 | 1.00 | 0.99 | 0.98 |
| C | 0704 | 10 | 10 | 1.00 | 1.00 | 1.00 | 1.00 |
| C | 0802 | 7 | 8 | 1.00 | 1.00 | 0.88 | 0.87 |
| C | 1202 | 10 | 10 | 1.00 | 1.00 | 1.00 | 1.00 |
| C | 1203 | 39 | 41 | 1.00 | 1.00 | 0.95 | 0.95 |
| C | 1205 | 1 | 0 | 0.00 | 1.00 | NA | 0.00 |
| C | 1213 | 1 | 0 | 0.00 | 1.00 | NA | 0.00 |
| C | 1402 | 10 | 10 | 1.00 | 1.00 | 1.00 | 1.00 |
| C | 1502 | 16 | 18 | 1.00 | 1.00 | 0.89 | 0.89 |
| C | 1504 | 1 | 0 | 0.00 | 1.00 | NA | 0.00 |
| C | 1505 | 6 | 5 | 0.83 | 1.00 | 1.00 | 0.83 |
| C | 1601 | 22 | 24 | 0.95 | 0.99 | 0.88 | 0.82 |
| C | 1604 | 3 | 0 | 0.00 | 1.00 | NA | 0.00 |
| C | 1701 | 7 | 14 | 1.00 | 0.99 | 0.50 | 0.49 |
| C | 1703 | 5 | 0 | 0.00 | 1.00 | NA | 0.00 |
|  |  |  |  |  |  |  |  |
| DQA | 0101 | 53 | 57 | 1.00 | 0.99 | 0.93 | 0.91 |
| DQA | 0102 | 108 | 111 | 0.94 | 0.97 | 0.91 | 0.80 |
| DQA | 0103 | 42 | 39 | 0.76 | 0.98 | 0.82 | 0.65 |
| DQA | 0104 | 2 | 0 | 0.00 | 1.00 | NA | 0.00 |
| DQA | 0105 | 1 | 0 | 0.00 | 1.00 | NA | 0.00 |
| DQA | 0201 | 86 | 87 | 1.00 | 1.00 | 0.99 | 0.99 |
| DQA | 0301 | 56 | 63 | 0.98 | 0.98 | 0.87 | 0.84 |
| DQA | 0302 | 3 | 0 | 0.00 | 1.00 | NA | 0.00 |
| DQA | 0303 | 6 | 0 | 0.00 | 1.00 | NA | 0.00 |
| DQA | 0401 | 18 | 19 | 1.00 | 1.00 | 0.95 | 0.94 |
| DQA | 0501 | 43 | 70 | 1.00 | 0.93 | 0.61 | 0.55 |
| DQA | 0505 | 26 | 0 | 0.00 | 1.00 | NA | 0.00 |
| DQA | 0509 | 1 | 0 | 0.00 | 1.00 | NA | 0.00 |
| DQA | 0601 | 1 | 0 | 0.00 | 1.00 | NA | 0.00 |
|  |  |  |  |  |  |  |  |
| DQB | 0201 | 82 | 82 | 1.00 | 1.00 | 1.00 | 1.00 |
| DQB | 0202 | 92 | 92 | 0.99 | 1.00 | 0.99 | 0.98 |
| DQB | 0301 | 139 | 138 | 0.99 | 1.00 | 0.99 | 0.97 |
| DQB | 0302 | 68 | 71 | 1.00 | 1.00 | 0.96 | 0.95 |
| DQB | 0303 | 27 | 29 | 1.00 | 1.00 | 0.93 | 0.93 |
| DQB | 0304 | 1 | 0 | 0.00 | 1.00 | NA | 0.00 |
| DQB | 0305 | 3 | 0 | 0.00 | 1.00 | NA | 0.00 |
| DQB | 0402 | 27 | 27 | 1.00 | 1.00 | 1.00 | 1.00 |
| DQB | 0501 | 78 | 77 | 0.97 | 1.00 | 0.99 | 0.96 |
| DQB | 0502 | 22 | 21 | 0.95 | 1.00 | 1.00 | 0.96 |
| DQB | 0503 | 16 | 17 | 1.00 | 1.00 | 0.94 | 0.95 |
| DQB | 0601 | 12 | 12 | 1.00 | 1.00 | 1.00 | 1.00 |
| DQB | 0602 | 110 | 115 | 1.00 | 0.99 | 0.96 | 0.95 |
| DQB | 0603 | 48 | 45 | 0.90 | 1.00 | 0.96 | 0.86 |
| DQB | 0604 | 26 | 25 | 0.96 | 1.00 | 1.00 | 0.96 |
| DQB | 0609 | 6 | 7 | 1.00 | 1.00 | 0.86 | 0.85 |
| DQB | 0614 | 1 | 0 | 0.00 | 1.00 | NA | 0.00 |
|  |  |  |  |  |  |  |  |
| DRB | 0101 | 134 | 116 | 0.84 | 1.00 | 0.97 | 0.82 |
| DRB | 0102 | 19 | 19 | 1.00 | 1.00 | 1.00 | 1.00 |
| DRB | 0103 | 12 | 30 | 0.75 | 0.99 | 0.30 | 0.22 |
| DRB | 0301 | 208 | 211 | 1.00 | 1.00 | 0.99 | 0.98 |
| DRB | 0302 | 1 | 0 | 0.00 | 1.00 | NA | 0.00 |
| DRB | 0401 | 130 | 184 | 0.98 | 0.96 | 0.70 | 0.66 |
| DRB | 0402 | 22 | 0 | 0.00 | 1.00 | NA | 0.00 |
| DRB | 0403 | 19 | 9 | 0.11 | 1.00 | 0.22 | 0.04 |
| DRB | 0404 | 51 | 65 | 0.92 | 0.99 | 0.72 | 0.65 |
| DRB | 0405 | 14 | 0 | 0.00 | 1.00 | NA | 0.00 |
| DRB | 0407 | 19 | 0 | 0.00 | 1.00 | NA | 0.00 |
| DRB | 408 | 4 | 0 | 0.00 | 1.00 | NA | 0.00 |
| DRB | 409 | 1 | 0 | 0.00 | 1.00 | NA | 0.00 |
| DRB | 410 | 1 | 0 | 0.00 | 1.00 | NA | 0.00 |
| DRB | 701 | 229 | 230 | 1.00 | 1.00 | 1.00 | 0.99 |
| DRB | 801 | 42 | 46 | 1.00 | 1.00 | 0.91 | 0.91 |
| DRB | 802 | 3 | 0 | 0.00 | 1.00 | NA | 0.00 |
| DRB | 810 | 1 | 0 | 0.00 | 1.00 | NA | 0.00 |
| DRB | 901 | 19 | 19 | 1.00 | 1.00 | 1.00 | 1.00 |
| DRB | 1001 | 17 | 16 | 0.94 | 1.00 | 1.00 | 0.94 |
| DRB | 1101 | 123 | 199 | 0.98 | 0.95 | 0.60 | 0.57 |
| DRB | 1102 | 3 | 4 | 0.67 | 1.00 | 0.50 | 0.33 |
| DRB | 1103 | 13 | 0 | 0.00 | 1.00 | NA | 0.00 |
| DRB | 1104 | 86 | 33 | 0.37 | 1.00 | 0.97 | 0.36 |
| DRB | 1106 | 1 | 0 | 0.00 | 1.00 | NA | 0.00 |
| DRB | 1115 | 1 | 0 | 0.00 | 1.00 | NA | 0.00 |
| DRB | 1139 | 1 | 0 | 0.00 | 1.00 | NA | 0.00 |
| DRB | 1143 | 1 | 0 | 0.00 | 1.00 | NA | 0.00 |
| DRB | 1201 | 27 | 25 | 0.93 | 1.00 | 1.00 | 0.92 |
| DRB | 1202 | 1 | 0 | 0.00 | 1.00 | NA | 0.00 |
| DRB | 1301 | 107 | 108 | 0.97 | 1.00 | 0.96 | 0.94 |
| DRB | 1302 | 72 | 72 | 0.96 | 1.00 | 0.96 | 0.91 |
| DRB | 1303 | 19 | 20 | 1.00 | 1.00 | 0.95 | 0.95 |
| DRB | 1305 | 4 | 0 | 0.00 | 1.00 | NA | 0.00 |
| DRB | 1315 | 1 | 0 | 0.00 | 1.00 | NA | 0.00 |
| DRB | 1401 | 40 | 45 | 1.00 | 1.00 | 0.89 | 0.84 |
| DRB | 1402 | 1 | 0 | 0.00 | 1.00 | NA | 0.00 |
| DRB | 1404 | 4 | 0 | 0.00 | 1.00 | NA | 0.00 |
| DRB | 1406 | 1 | 0 | 0.00 | 1.00 | NA | 0.00 |
| DRB | 1501 | 205 | 207 | 1.00 | 1.00 | 0.99 | 0.99 |
| DRB | 1502 | 22 | 21 | 0.95 | 1.00 | 1.00 | 0.95 |
| DRB | 1601 | 49 | 51 | 1.00 | 1.00 | 0.96 | 0.96 |
| DRB | 1602 | 2 | 0 | 0.00 | 1.00 | NA | 0.00 |

Allele-specific sensitivity, specificity, PPV and r^2^ for the first experiment (HLA*IMP:02, GS -> HLARES_EU). “NValidation” specifies how often an allele appears in the validation data (according to classical typing results, which we treat as the truth in this experiment). “NImputation” specifies how often an allele appears in the imputations for the validation data. The following columns specify sensitivity, specificity, PPV and r^2^ for each allele. All numbers are based on “best-guess” called alleles.
