# Supplementary material for: Multi-Population Classical HLA Type Imputation
Source: PLoS Comput Biol. 2013 Feb 14;9(2):e1002877. doi: 10.1371/journal.pcbi.1002877 (PMC3572961; doi:10.1371/journal.pcbi.1002877)
Supplement: Text S1 — The HLA*IMP:02 model and algorithms. Mathematical and algorithmic characterization of the haplotype graph model of HLA*IMP:02, allowing for integrating over path uncertainty and localization. (PDF) [file pcbi.1002877.s011.pdf]

Multi-population classical HLA type imputation
Supporting Text S1

Alexander Dilthey * [1,2,†], Stephen Leslie[3,†], Loukas Moutsianas[2], Judong Shen[4], Charles Cox[5], Matthew R. Nelson[4], Gil McVean[1,2]

[1]Department of Statistics, University of Oxford, 1 South Parks Road, Oxford, OX1 3TG, United Kingdom
[2]Wellcome Trust Centre for Human Genetics, Roosevelt Drive, Oxford OX3 7BN, United Kingdom
[3]Murdoch Childrens Research Institute, Royal Children's Hospital, Flemington Road, Parkville, Victoria 3052 Australia
[4]Quantitative Sciences, GlaxoSmithKline, Research Triangle Park, NC 27709, USA
[5]Quantitative Sciences, GlaxoSmithKline, Stevenage, UK
[†]contributed equally
* dilthey@well.ox.ac.uk

Before describing the algorithmic details of HLA*IMP:02, we give a high-level overview. In genotype imputation, a reference panel with high marker density and a statistical model of population haplotype structure are used to impute missing markers in imputation panels with lower marker density [1]. The situation we deal with in this paper can be described as follows: suppose there is a reference panel R, consisting of the genotype data G_R of N_R individuals, typed at a (non-empty) set of loci L on the same chromosome. Also, there is an imputation panel I, consisting of the genotype data G_I of N_I individuals, typed at a (non-empty) set of loci L' ($L' \subseteq L$). The algorithm we present here then

1. uses R to construct a model of the haplotype structure of the haplotypes present in R. We denote this model as M, and it will, like many other models in population genetics, assign a likelihood to every possible haplotype over the L loci. M belongs to the class of haplotype graph models (formally described below).

2. treats each individual in I independently, and makes inference on the loci of interest $\{L \setminus L'\}$ by integrating over possible underlying haplotypes. That is, for the i-th individual in I, we evaluate the conditional probability $\mathrm{P}((h_1, h_2) \mid G_{I,i}, M)$, where M is our model of haplotype structure, $G_{I,i}$ the genotype data of the i-th individual I_i in I and (h_1, h_2) a pair of haplotypes for I_i at the specified L loci. Each (h_1, h_2) implies a genotype at all loci we may want to consider, and we average over these according to $\mathrm{P}((h_1, h_2) \mid G_{I,i}, M)$. Note that no information from I is used to build M.

In the following, we will therefore describe two separate tasks: how to infer a haplotype graph model M from a set of genotyped individuals and how to use this model to infer the missing genotypes of an individual with genotype $G_{I,i}$. Note that, although we use a haplotype structure model, haplotype inference (phasing) is not our aim in this paper: we are only interested in the correctness of the resulting genotype imputations. Note also that we will only make explicit reference to HLA types during later stages – for now, we assume that a locus may have an arbitrary number of alleles, and do therefore not fundamentally differentiate between a SNP and a classical HLA locus.

Note that Figures 1, 3 and 2 in this document provide a compressed view of the algorithm's most important features.

Haplotype graph models and their induced HMMs

We give formal definitions of haplotypes, haplotype graph models and how they relate to HMMs.

We define haplotypes to be strings of symbols of length $T = |L|$ (therefore $T > 0$), where at any given position $p \in \{0 .., (T-1)\}$ the symbols come from some predefined set A_p, the *model alphabet*. In the context of genetics the model alphabet may be, for example, the set of possible nucleotides (A, C, G and T).

Let the directed connected graph M consist of the directed edges E and the vertices V, i.e. $M = (V, E)$. If $v_a, v_b \in V$ then we define (v_a, v_b) to be the edge in E running from v_a to v_b, provided such an edge exists, and we say $(v_a, v_b) \in E$. For haplotype graphs, each vertex $v \in V$ has an associated well-defined level function $l(v)$, according to the following definition. There is exactly one vertex v_0 with no incoming edges and $l(v_0) = 0$. This vertex is called the root vertex. For every $(v_a, v_b) \in E$, we define $l(v_b) = l(v_a) + 1$. All vertices with no outgoing edges are called "final vertices", and in the case of haplotype graphs all such vertices have the same level, T. Each level can be thought of as a genetic locus. At each level p there is a set of possible emission symbols, the "model alphabet" A_p. Each edge (v_a, v_b) at level p (i.e. $l(v_a) = p$) has an associated emission symbol from A_p. There are no two edges with the same emission symbol originating from the same vertex. Each edge has an associated transition probability, and the transition probabilities of all edges emanating from a non-final vertex add up to 1.

Haplotype graphs probabilistically generate strings of the same length (haplotypes). We give a description of the algorithm that produces haplotypes from a haplotype graph model. Begin at vertex v_0. If at vertex v_a, select one outgoing edge $e = (v_a, v_b)$ according to the probabilities attached to the edges emanating from v_a. Emit the symbol attached to e. Move to the "target vertex" v_b. Continue this procedure at v_b until v_b is a final vertex with no outgoing edges. This may be thought of as defining a *path* through the graph, i.e. a connected sequence of vertices from v_0 to a final vertex at level T. Note that all so-generated haplotypes have the same length.

Browning and Browning [2] note that haplotype graph models as described above are Hidden Markov Models (HMMs). Each edge corresponds to a state, and the transition

probabilities between any two edges (v_a, v_b) and (v_c, v_d) are 0 unless $v_c = v_b$. If $v_c = v_b$ then the transition probabilities are defined by the edge probability distribution at v_b. In a basic model, for a given state the emitted symbol (e.g. nucleotide) is just the symbol associated with the corresponding edge.

The induced HMM so-described provides a haploid model for genetic data. The generalization for diploid data, based on two connected haploid HMMs and their combined emission probabilities follows immediately, as described by Browning and Browning [2]. Informally, if the haploid model has n states mapping to level p in the haplotype graph, the diploid model has n^2 states at the same level, mapped to the set of ordered pairs (k, r) $(k \in \{1..n\},\ r \in \{1..n\})$. To be explicit, state (k, r) refers to the first of the two connected haploid models being in state k, and the other one being in state r. In a basic emission probability distribution, (k, r) emits the unordered 2-tuple (genotype) of the emission symbols associated with the haploid states k and r, and more complex emission probability distributions follow from combining the respective haploid emission probability distributions. The transition probability of (k, r) at level p to (k', r') at level $p+1$ is equal to the product of the haploid transition probabilities from k to k' and r to r'.

Note that at this point we can sample diploid (i.e., sequences g of T ordered genotypes) and haploid (i.e., haplotypes h) data from the graph-equivalent HMMs: $\mathrm{P}(h \mid M \to \mathrm{HMM}_1)$ and $\mathrm{P}(g \mid M \to \mathrm{HMM}_2)$ are both well-defined ($M \to \mathrm{HMM}_1$ denotes the M-equivalent haploid HMM, and $M \to \mathrm{HMM}_2$ denotes the M-equivalent diploid HMM). If we have observed haploid data h, we can use standard statistical techniques [3] to sample a haplotype h' (formed from concatenating the emission symbols of the edges associated with the traversed states) from $\mathrm{P}(h' \mid h, M \to \mathrm{HMM}_1)$. Both h and h' are haplotypes of length T, so that this expression does not seem to be very useful. Now, suppose that h was actually only typed at the loci specified by L', and that all other loci carry a "missing data" symbol E_M. If we now modify the emission probability structure of $M \to \mathrm{HMM}_1$ accordingly, for example by assigning each state the same probability to emit E_M (we say that this is an "agnostic" way to deal with missing data), we can use samples from $\mathrm{P}(h' \mid h, M \to \mathrm{HMM}_1)$ to estimate symbols for the positions carrying an E_M in h. This property immediately translates to $M \to \mathrm{HMM}_2$, the M-equivalent diploid HMM. That

is, conditional on some genotype data g for an individual, with some of the positions potentially being missing data, we can sample from $\mathrm{P}((h_1, h_2) \,|\, g, M \rightarrow \mathrm{HMM}_2)$ ((h_1, h_2) is a pair of two haplotypes, formed from concatenating the emission symbols of the pairs of edges associated with the traversed states). If desired, by marginalizing over the respective elements in (h_1, h_2), it is now possible to independently estimate the underlying genotype of any of the loci in L loci conditional on M and g, including of course the loci $L \setminus L'$.

We have now described a (well-known, see [2]) solution to the second task: inference of missing genotypes for an additional individual, conditional on some observed genotype data and a haplotype graph M. By specifying the emission probability structure of $M \rightarrow \mathrm{HMM}_1$ in a way that allows for emitting other symbols than E_M or the state's underlying emission symbol, we introduce a mutation- or error-like effect (like [4]). For all following applications, we define a *graph sampling error* parameter m_S for the emission probability structure of haplotype graph-induced HMMs: conditional on not emitting E_M, the state's underlying emission symbol (coming from the associated edge in M) is emitted with probability $1 - m_S$. With probability m_S, one of the other members of the locus-specific model alphabet is uniformly selected and emitted.

Constructing a haplotype graph

We now describe our algorithm to construct a haplotype graph model M from a set R of individuals genotyped at T loci.

Note that the following description is conceptual – when actually implementing the algorithm, we employ a couple of heuristics to reduce the computational effort (see Section "Computational efficiency" for details).

Following [2], we employ an iterative strategy, with Z_{stop} (the number of iterations) usually set to 12:

1. Define a set H of temporary haplotypes and populate H with a number of samples for each individual i in R. The samples are generated by drawing N_S times from a uniform probability distribution over all pairs of haplotypes which are compatible (ignoring read error or mutation) with an individual's genotypes $G_{R,i}$, and each haplotype pair is broken up into two separate haplotypes before insertion into H.

Missing data in the genotypes is carried over to the haplotypes. Set $Z := 1$.

2. Construct a haplotype graph model M based on H, as described below.
3. Set $H = \{\}$. For each reference individual i, draw N_S samples from $\mathrm{P}((h_1, h_2) \,|\, G_{R,i}, M \rightarrow \mathrm{HMM}_2)$, and add h_1 and h_2 to H.
4. Invert each haplotype in H.
5. Set $Z := Z + 1$, terminate if $Z > Z_{\mathrm{stop}}$. Otherwise, go to step 2.

Our haplotype graph construction algorithm is a probabilistic generalization of the works of Browning and Browning [2], which allows for uncertainty and missing data in the set of estimated haplotypes H and a tailoring of the graph according to prior knowledge on regional LD structure ("localization"). The aim is to infer an accurate and computationally convenient haplotype graph model from the set of haplotypes H.

Suppose for a given number of levels T (corresponding to the lengths of the haplotypes in the set H) we have the most general possible haplotype graph topology, i.e. a tree for which every possible emission symbol has an edge at every vertex in the graph. Note that at this stage there are no probabilities assigned. Each $h \in H$ with no missing data corresponds to a unique path through the graph topology, and we say that h is *attached* to all vertices that the path passes through. However, we want to allow for missing data in H, and we also want to take into account the possibility that an error process may have modified the elements in H prior to observation. In the context of genetics, it is easy to see why this makes sense. For example, a SNP genotyping error may lead to a haplotype being present in H which does really not exist in the population.

We define a simple error process for the elements in H. We assume that this error process acts independently on each character position and that, if an error occurs (with probability m_B), a new observed value is drawn from a uniform distribution over possible alternative alleles at the affected position (this could, if desired, be easily generalized to more complex error models). If we observe string h_1 of length T, the likelihood that string

h_2 is the true underlying string is

$$\prod_{p=0\,..\,T-1} \left[I_{h_1(p)==h_2(p)} \times (1-m_B) + (1-I_{h_1(p)==h_2(p)}) \times \frac{m_B}{|A_p|-1}\right],$$

where we define $I_{h_1(p)==h_2(p)}$ to be 1 if the p-th symbol of h_1 is equal to the p-th symbol of h_2 and 0 otherwise. For simplicity, although m_B may capture other effects than error, we refer to m_B as the *graph building error* probability. $|A_p|$ is the number of available symbols at haplotype position p (the size of the model alphabet at p).

If we observe missing data, we want to treat it in an agnostic way, i.e. assume equal probabilities for each symbol in the model alphabet at the corresponding position.

We now probabilistically attach the haplotypes in H to the most general possible haplotype graph topology for T levels (in our implementation, we actually prune the tree as we move along the haplotypes – see Section "Computational efficiency" for details). For each vertex, we introduce a list of probability-weighted potentially attached haplotypes. At each level of the graph, the sum of attachment probabilities has to be 1 for each haplotype. All haplotypes are attached to the root vertex with probability 1 by defining the attachment probability $\mathrm{P}_H(v_0, h) := 1$ for all $h \in H$; they are then distributed along the graph according to our error model. That is, if haplotype h is attached to v_a at level $l(v_a)$ with probability y, and if the next observed haplotype symbol is $s \neq E_M$, we have the following attachment probabilities for the children v_b of v_a at level $l(v_a)+1$: if the edge (v_a, v_b) carries the attached symbol s, the attachment probability of h at v_b is $y \times (1-m_B)$, i.e. we define $\mathrm{P}_H(v_b, h) := \mathrm{P}_H(v_a, h) \times (1-m_B)$. Otherwise, the attachment probability is $y \times \frac{m_B}{|A_{l(v_a)}|-1}$, and we define $\mathrm{P}_H(v_b, h) := \mathrm{P}_H(v_a, h) \times \frac{m_B}{|A_{l(v_a)}|-1}$. If $s = E_M$, we attach h in an agnostic manner, i.e. we define $\mathrm{P}_H(v_b, h) := \mathrm{P}_H(v_a, h) \times \frac{1}{|A_{l(v_a)}|}$.

For notational convenience, let $\mathtt{attached}(v)$ denote the set of haplotypes attached to v with attachment probability $\mathrm{P}_H(v, h) > 0$. To examine the structure of the graph topology with attached haplotypes, for each vertex v, we define a function $\mathtt{count}(v, x)$. If x is the empty string $''$, $\mathtt{count}(v, x)$ returns the expected number of haplotypes in H attached to v:

$$\texttt{count}(v,'') = \sum_{h \in \texttt{attached}(v)} \mathrm{P}_H(v,h)$$

If x is a string of length ≥ 1, $\texttt{count}(v,x)$ returns the expected number of haplotypes that continue with a specified suffix x of length $\texttt{len}(x) \geq 1$ to the right-hand side of v. x can be a partial or complete suffix, i.e. of length $1..[T - l(v)]$:

$$\texttt{count}(v,x) = \sum_{h \in \texttt{attached}(v)} \Big(\mathrm{P}_H(v,h) \times \prod_{p=[l(v)]..[l(v)+\texttt{len}(x)-1]} \big[I_{h(p)==x(p)} \times (1 - m_B) + (1 - I_{h(p)==x(p)}) \times \frac{m_B}{A_p - 1}\big]\Big)$$

We complete the definition of a haplotype graph by specifying edge transition probabilities. Define $\mathrm{P}(e|v)$ as the probability to follow edge e conditional on being at vertex v, and let s denote the symbol that is attached to e. Then we set

$$\mathrm{P}(e|v) := \texttt{count}(v,s)/\texttt{count}(v,'')\,,$$

Figure 1 in this document illustrates the effect of the described algorithm: instead of taking one specified path through the graph topology, a haplotype's probability "flows" through the graph.

However, we observe i) that the resulting haplotype graph exhibits a considerable topological complexity, if built from a reasonably sized set H, possibly leading to computational difficulties in later stages and ii) that the topology of the graph is still the most general one. If we assume that H was actually sampled from a haplotype graph, and if we want to recover the original graph's underlying structure, we have to take into account the possibility that the original graph's structure may have been simpler, i.e. that one vertex in the original graph corresponds to more than one vertex on the same level in the current graph. Introducing a criterion of similarity that is based on comparing vertices' conditional suffix distributions addresses both points. Informally speaking, vertices with very similar suffix output distributions can be merged into one vertex to reduce computa-

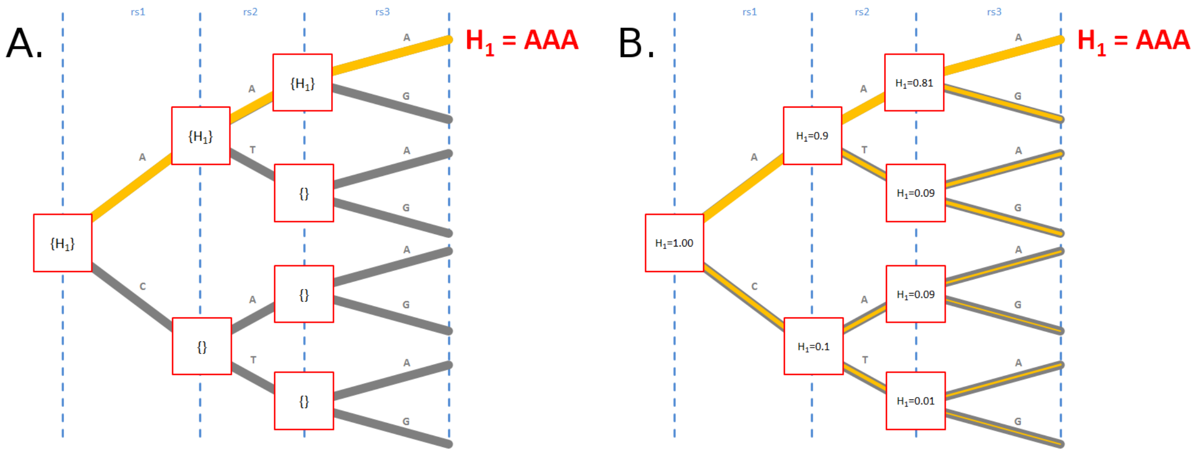

Figure 1: A: a non-probabilistic haplotype graph construction algorithm. Each haplotype in the set H follows one defined path (orange) through the graph's possible topology (orange and gray branches), here depicted for $H_1 = \texttt{AAA}$. Each node (red squares) carries a list of attached haplotypes. B: the probabilistic haplotype graph construction algorithm presented in this chapter. Each haplotype in the set H induces a probability distribution over possible paths through the graph, here pictured as orange lines. The width of the lines indicates how probable a path is according to the path probability distribution (not drawn to scale). At each node, the path follows the edge carrying the haplotype's next symbol with probability $1 - m_B$, and the remaining probability mass is split over the remaining available edges. Each node carries a list of attached haplotypes with the respective attachment probability. The figure is based on a path distribution for "AAA", with the graph-building error probability m_B set to 0.1.

tional demands without substantially changing the model's haplotype frequencies. Also, if two vertices were actually identical or not distinguishable in an original haplotype graph model, we would expect their suffix output distributions to be similar (see [5] for a formal treatment).

We formalize the notion of similar suffix distributions following Ron et al. [5] and Browning and Browning [2] by defining the function $\texttt{similar}(v_a, v_b)$ as the maximum difference between the two conditional suffix probability distributions of v_a and v_b:

$$\texttt{similar}(v_a, v_b) := \max_{x \in S_{v_a, v_b}} \left| \mathrm{P}_{\text{Suffix}}(v_a, x) - \mathrm{P}_{\text{Suffix}}(v_b, x) \right| ,$$

where we define

$$\mathrm{P}_{\text{Suffix}}(v, x) := \texttt{count}(v, x) / \texttt{count}(v, '') .$$

S_{v_a, v_b} is defined as the set of possible suffixes originating from v_a or v_b (partial or complete). In order to accommodate the complex haplotype structure of the MHC, we

include the edge label leading to a node as the first character of all suffixes.

We apply `similar` to all pairs of vertices (v_a, v_b) at all levels to identify pairs of vertices that can be merged. If $\texttt{similar}(v_a, v_b) < \epsilon$, two vertices are merged. We follow Browning and Browning [2] in using a variance-based threshold:

$$\epsilon := D \times \sqrt{N_S/2} \times (\texttt{count}(v_a,'') ^{-1} + \texttt{count}(v_b,'')^{-1})^{1/2} \,,$$

where D is a scale parameter (usually 0.8 here, determined by initial experiments) and N_S is the number of haplotype pair samples from each individual.

To merge v_a and v_b,

1. create a new vertex v_c at the same level as v_a and v_b
2. redirect all incoming edges of v_a and v_b to v_c, and for all $h \in \{\texttt{attached}(v_b) \cup \texttt{attached}(v_a)\}$, set $\mathrm{P}_H(v_c, h) := \mathrm{P}_H(v_b, h) + \mathrm{P}_H(v_a, h)$
3. attach all outgoing edges of v_b and v_a to v_c, and delete v_b and v_a.
4. note that step 2 will result in a structure violating the haplotype graph assumptions, as it will result in two edges (v_c, v_d) , $(v_c, v_{d'})$ with the same attached symbol. Merge v_d and $v_{d'}$ as described for all such cases (i.e. recursively from step 1, if necessary), and delete one of the two resulting edges leading to the new node replacing d and d'.
5. finally, update $\mathrm{P}(e|v)$ for all modified vertices and compute the similar function for v_c and all other vertices on the same level.

Figure 2 in this document illustrates the process of merging nodes. For notational convenience, we have assumed a fixed graph building error probability m_B here for all loci, but it is easy to see that this is not necessary.

Finally, we describe how to localize the graph construction process. Localization aims at incorporating prior knowledge on patterns of long-range LD into the graph-building process. Consider the following example to see why localization can be sensible. Suppose that two haplotypes from H are attached to v_a: '00A' and '11A' (the allele identifiers are arbitrary). Suppose further that a node v_b on the same level has also two attached

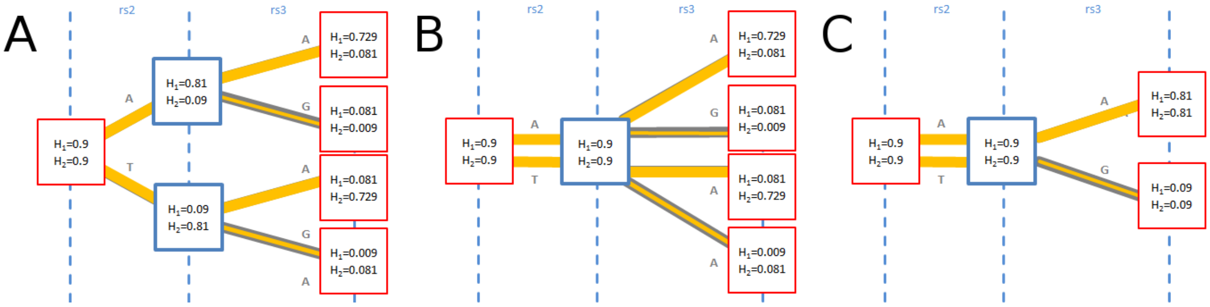

Figure 2: The essential steps of merging nodes in the probabilistic framework described here. A: two haplotypes (AAA and ATA) have been attached to the topology shown in Figure 1 in this document (the graph's first level is not shown) with $m_B = 0.1$. The conditional suffix distributions of two nodes (pictured as blue squares) are identical and the nodes will be merged. B: all outgoing edges from the two nodes have been attached to one newly created joint node (blue square). The resulting structure is no haplotype graph, because two edges emanating from the new node carry the same symbols as two other edges emanating from the same node. C: The nodes that the conflicting edges lead two are recursively merged, resulting in a haplotype graph structure.

haplotypes, '00B' and '11B'. If we compare the conditional suffix distributions, we find no difference for suffixes of length up to 2. For suffixes of length 3 and a small m_B, we find that the maximum difference is just below 0.5 (because none of the 3-character suffixes present in one vertex is present in the other one). Depending on our choice of ϵ, we may decide to merge the two vertices. The problem here is that the vertices actually exhibit quite different patterns of LD to the third position – the maximum conditional probability difference is almost 1. The localization element extends the function `similar` to take into account such situations for a set S_L of levels of predefined loci and could therefore prevent merging the two vertices.

Define the indicator function $I_{h(p)==s}$ to be 1 if haplotype h carries allele s at position p, and 0 otherwise. We define

$$\text{P}_{\text{LOCALIZE}}(v, s, p) := \frac{\sum_{h \in \texttt{attached}(v)} \text{P}_H(v,h) \times (I_{h(p)==s} \times (1-m_B) + (1-I_{h(p)==s}) \times \frac{m_B}{|A_p|-1})}{\texttt{count}(v,'')}.$$

We note that this conditional probability integrates over the uncertainty in the intermediate SNP genotypes and redefine the `similar` function to include all loci specified in S_L:

$$
\begin{aligned}
&\texttt{similar}(v_a, v_b) := \max \{ \\
&\max_{x \in S_{v_a, v_b}} |\mathrm{P}(v_a, x) - \mathrm{P}(v_b, x)|, \\
&\max_{\{p \in S_L, s \in A_p\}} |\mathrm{P}_{\textsc{Localize}}(v_a, s, p) - \mathrm{P}_{\textsc{Localize}}(v_b, s, p)| \\
&\}.
\end{aligned}
$$

Figure 3 in this document illustrates the localization feature.

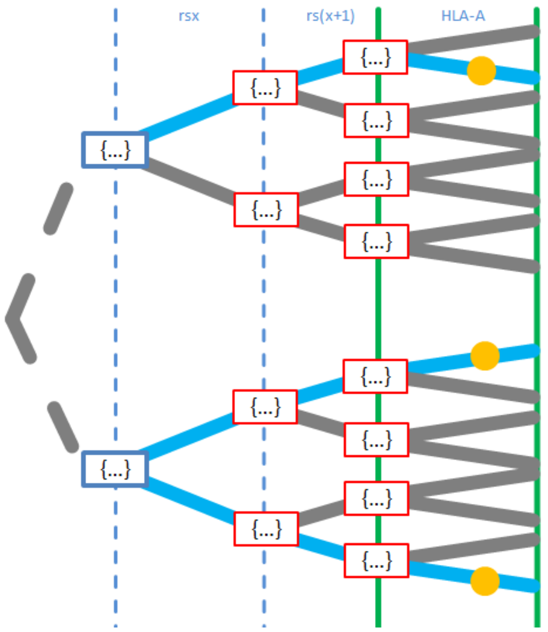

Figure 3: Localization at the example of an HLA locus. When comparing the conditional HLA allele probabilities for two nodes (blue squares) for a particular *HLA-A* allele (marked with an orange circle in the graph), the probabilities of all paths leading to this allele are added up (separately for each node). Note that the two blue paths for the lower node would count as two distinct suffixes without localization.

Computational efficiency

The algorithm we have described to build localized haplotype graphs from an uncertain set of haplotypes requires substantial computational resources: to calculate the conditional suffix distributions for each vertex, it is necessary to sum over all attached haplotypes with attachment probability > 0. Haplotype attachment distributions of single haplotypes are

typically (depending on the uncertainty model and the number of alleles at the involved loci) very skewed: a few vertices at any level usually account for most of the available probability. Therefore, a threshold t is introduced: if $\mathrm{P}_H(v,h) < t$, $\mathrm{P}_H(v,h)$ is set to 0 and the removed probability mass is spread proportionally over all vertices with $\mathrm{P}_H(v,h) \geq t$. Also, when computing $\texttt{similar}(v_a, v_b)$ for two vertices, only such $x \in S_{v_a,v_b}$ that are present in at least one of the haplotypes attached to v_a or v_b are evaluated – suffixes x merely induced by error processes on both vertices will carry smaller probabilities than the original strings and therefore lead to a smaller absolute difference in probability.

HLA type inference

HLA loci are treated as multi-allelic SNPs, i.e. the observed HLA types are part of the haplotype strings H and appear as edges in the haplotype graph. SNP- and HLA-genotyped individuals can be used as input for the "iterative refinement" algorithm, without prior phasing. Only individuals with at least one genotyped 4-digit HLA allele are used for constructing the haplotype graph model, and 4- and 2-digit alleles are treated in the same way, i.e. as unrelated, separate entities (4-digit resolution specifies the primary structure of the classical HLA proteins, whereas 2-digit resolution refers to more general serological properties of the alleles).

To account for the long-range LD structure of the MHC region, the graph building algorithm is localized for all classical HLA loci but *B* and *DRB1* (see below). Usually, we set $m_B = m_S$.

Note that the results from step 3 of the model building algorithm can be used to quantitatively assess whether a lab-based HLA typing result in the reference dataset is consistent with the graph or not; the posterior probabilities follow from summing over the haplotype samples. To minimize the impact of mis-typed HLA alleles in the reference panel, after a specific number of graph-building and sampling iterations (usually 8), the number of sampled haplotypes for a specific individual is weighted by the internally estimated probability that the individual's lab-based HLA type is consistent with the graph.

We build locus-specific HLA haplotype graphs for windows of a specified size each side of the locus; 300 SNPs each side have been found to give good results. HLA type inference

is carried out by sampling haplotype pairs from the diploid HMM, conditional on the observed genotypes $G_{I,i}$ for each individual i in the inference dataset and the haplotype graph: $\mathrm{P}((h_1, h_2) \,|\, G_{I,i}, M \to \mathrm{HMM}_2)$. This leads to posterior distributions over possible pairs of HLA types that can be processed in an uncertainty-aware way or thresholded. To call alleles, we first determine the most likely single allele for each individual and then the most likely second allele, conditional on that individual carrying the first allele. We use the marginal probability to observe the first allele (i.e. summed over all samples from the haplotype pair distribution) as quality score ("allele-specific posterior probability") for the first allele and the joint probability for the first and the second allele as quality score for the second allele.

Properties of the presented model and parameter inference

We have presented a generalized haplotype graph construction algorithm, related to the BEAGLE algorithm [2] and earlier work in computational linguistics [5], which probabilistically attaches haplotypes to vertices while building the graph. We have introduced two additional parameters: a graph building error parameter m_B and the set of localization loci S_L that can be used to adapt graph construction to complex patterns of LD. Our algorithm also allows for missing data.

We briefly discuss some properties of the generalized model:

- The error model we have introduced leads to a relative decline of the importance of long-range haplotype differences in terms of collapsing vertices: $|P_{\mathrm{Suffix}}(v_1, x) - P_{\mathrm{Suffix}}(v_2, x)|$ is decreased for x with large differences. This depends on d, the scaling parameter in the collapsing criterion, and m_B, the error probability.

- We expect the generalized model to be potentially useful in other applications than the one considered here. For example, if a haplotype graph is to be constructed for a set of experimentally determined haplotypes (from single chromosome sequencing, say), the uncertainty model for the graph-building step we have introduced can be used to model read errors.

- The described algorithm can deal with missing data in the set of haplotypes in a

straightforward way by defining a probability distribution on missing characters, e.g. a uniform distribution. This property allows us not having to guess genotypes for the first iteration of graph-building. Although the algorithm as described here imputes missing genotypes in the reference panel during the first sampling process, the missing data status could as well be preserved in the sampled haplotypes and could be carried over to later stages. As the reference panels we are dealing with are consistently typed on dense sets of markers, we have decided against this possibility here. However, under other circumstances, for example when SNP coverage in the reference panel varies strongly, not imputing missing SNP data may turn out to be beneficial [6].

- Treating HLA alleles as multiallelic SNPs leads to a couple of useful properties in learning and inference settings. The graph itself can reflect patterns of long-range linkage disequilibrium between HLA alleles – HLA and SNP genotypes are used to infer the graph structure in a combined manner, and there is no requirement that all individuals be typed at the same set of HLA loci. Consider, for example, an inference dataset with *HLA-DRB1*-typed individuals, but lacking information for *HLA-DQB1*. Providing the *DRB1* genotypes as well as the SNP genotypes enables the model to use partial HLA type information in inferring missing bits of the complete HLA type (depending on the particular structure of the graph used for inference, of course).

Choosing optimal parameters for building haplotype graphs and for inference is an important direction for further research. For the experiments presented in the main text, we have used: $m_B = m_S = 0.002, \quad t = 0.001, \quad N_S = 50, \quad D = 0.8\,.$

Although standard statistical techniques like Maximum Likelihood and Markov Chain Monte Carlo could be applied in theory, the computational costs to do so seem prohibitive at the moment. In the context of this paper, our main purpose is statistical HLA type imputation, and we measure the fit of model and parameterization by the validation experiments presented in the main text. In order to justify the introduction of additional parameters, we have repeated some of the experiments presented in Leslie et al. [7] and Dilthey et al. [8]. We have used CEU HapMap data as a reference panel, constructed haplotype graphs and imputed HLA types into a subset of the BC58 (all data exactly as

described in our earlier papers). The results are summarized in Table S1. The column “HLA*IMP:02” refers to the full model (with parameters adapted to accommodate the much reduced panel size). In column I, the error probabilities for sampling from the graph and for building the graph are set to 0 (all other parameters equal to the full model). In column II, the error probability for building the graph is set to 0, and in column III, the error probability for sampling from the graph is set to 0. We find that the full model outperforms each of the reduced versions. In column IV, we have deactivated HLA localization. Interestingly, the full model only yields better results at *A* and *DQB1*, whereas the results at *B* and *DRB1* are worse. This may relate to classical typing problems, potentially associated with hypervariability (*B*) and nearby structural variation (*DRB1*), or it may indicate that localization does not improve imputation accuracy. Until further investigation, we deactivate localization for *B* and *DRB1*.

References

1. Marchini J, Howie B (2010) Genotype imputation for genome-wide association studies. Nat Rev Genet 11: 499-511.

2. Browning SR, Browning BL (2007) Rapid and accurate haplotype phasing and missing-data inference for whole-genome association studies by use of localized haplotype clustering. Am J Hum Genet 81: 1084-97.

3. Rabiner LR (1989) A tutorial on hidden Markov models and selected applications in speech recognition. Proceedings of the Ieee 77: 257-286.

4. Browning BL, Yu Z (2009) Simultaneous genotype calling and haplotype phasing improves genotype accuracy and reduces false-positive associations for genome-wide association studies. Am J Hum Genet 85: 847-61.

5. Ron D, Singer Y, Tishby N (1998) On the learnability and usage of acyclic probabilistic finite automata. Journal of Computer and System Sciences 56: 133-152.

6. Howie BN, Donnelly P, Marchini J (2009) A flexible and accurate genotype imputa-

tion method for the next generation of genome-wide association studies. PLoS Genet 5: e1000529.

7. Leslie S, Donnelly P, McVean G (2008) A statistical method for predicting classical HLA alleles from SNP data. Am J Hum Genet 82: 48-56.

8. Dilthey AT, Moutsianas L, Leslie S, McVean G (2011) HLA*IMP – an integrated framework for imputing classical HLA alleles from SNP genotypes. Bioinformatics 27: 968-72.
